# Effects of low-frequency and high-frequency electroacupuncture pretreatment on the COX-2/mPGES-1/PGE2 pathway in a rat model of cold-coagulation dysmenorrhea

**DOI:** 10.3389/fimmu.2025.1563626

**Published:** 2025-06-04

**Authors:** Yue Song, Jinxia Ni, Jingxue Yuan, Ziniu Zhang, Dinghao Wang, Zhihao Xiong

**Affiliations:** Dongzhimen Hospital, Beijing University of Chinese Medicine, Beijing, China

**Keywords:** low-frequency electroacupuncture, high-frequency electroacupuncture, dysmenorrhea, β-EP, COX-2/mPGES-1/PGE2 pathway

## Abstract

Primary dysmenorrhea (PD) refers to a condition in which women experience cyclic lower abdominal pain, often accompanied by lower back pain, and even severe pain and fainting around menstruation, significantly affecting daily activities and work. Inflammatory factors such as prostaglandins PGF2α, PGE2, and COX-2 play key roles in the pathological mechanisms of PD. Nonsteroidal anti-inflammatory drugs (NSAIDs) and hormonal contraceptives are the first-line treatments for dysmenorrhea; however, they have significant side effects. Electroacupuncture, as a non-invasive and effective therapeutic method, is widely used as a complementary and alternative treatment, although its mechanism of action remains unclear.

**Methods:**

A cold-coagulation type dysmenorrhea model was established in rats, which were divided into the control group, model group, low-frequency electroacupuncture group (2/10 Hz), and high-frequency electroacupuncture group (20/100 Hz). Behavioral writhing responses, organ indices, uterine tissue morphology changes, and levels of PGF2α, PGE2, COX-2, and other inflammatory markers were measured to evaluate the effects of the treatments.

**Results:**

Both the low-frequency electroacupuncture group and the high-frequency electroacupuncture group significantly reduced writhing scores, increased spleen index, decreased prostaglandin PGF2α and COX-2 levels, and increased prostaglandin PGE2 and β-EP levels to varying degrees.

**Discussion:**

Both low-frequency and high-frequency electroacupuncture exert their anti-inflammatory and analgesic effects by activating the COX-2/mPGES-1/PGE2 pathway and mediating the increased release of the opioid peptide β-EP, which alleviates inflammatory pain. Both treatments achieve the therapeutic goal of treating PD, with high-frequency electroacupuncture demonstrating superior anti-inflammatory and analgesic effects compared to low-frequency electroacupuncture.

## Introduction

Dysmenorrhea refers to the occurrence of periodic lower abdominal pain, often accompanied by lower back pain, and in severe cases, can lead to fainting, thereby affecting a woman’s normal work and daily life during menstruation or the days surrounding it. Clinically, dysmenorrhea is categorized into Primary Dysmenorrhea (PD) and Secondary Dysmenorrhea (SD) based on the presence or absence of organic pelvic and genital organ lesions (1). The global prevalence of PD ranges from 50% to 90%, making it one of the most common gynecological disorders (2), and its incidence has been on the rise over the past decade (3). Dysmenorrhea negatively impacts women’s quality of life (4). Current clinical guidelines for dysmenorrhea (5) recommend Nonsteroidal Anti-inflammatory Drugs (NSAIDs) and hormonal contraceptives as first-line treatments; however, these medications can produce side effects on the nervous and gastrointestinal systems (6). Both clinical trials and animal studies have demonstrated the efficacy and reliability of acupuncture in treating inflammatory pain (7). Consequently, electroacupuncture, an important modality of acupuncture therapy, possesses anti-inflammatory effects (8, 9).

The pathogenesis of PD remains not fully elucidated, but modern medicine largely attributes it to the synthesis and release of prostaglandins, which generate inflammatory responses leading to dysmenorrhea (7). Prostaglandin F2α (PGF2α) and Prostaglandin E2 (PGE2) play crucial roles in the occurrence and development of PD (1, 10). Studies have shown that PGF2α and PGE2 act through cyclooxygenase (COX) enzymes (11, 12), resulting in increased uterine tension and spasmodic contractions, which cause uterine ischemia and hypoxia, thereby inducing dysmenorrhea (13–15). In contrast to PGF2α, PGE2 has opposing effects in the development of PD; PGE2 can dilate blood vessels, inhibit uterine smooth muscle contractions, and reduce spontaneous uterine activity (16–18). β-Endorphin (β-EP) is an endogenous neuromodulator primarily produced and stored in the anterior pituitary gland of the brain (19, 20). As an endogenous opioid peptide, β-EP regulates endocrine function, stress responses, and analgesic effects, primarily participating in the modulation of pain signaling at the spinal cord level and other sites (21). Elevated levels of β-EP initiate the expression of endogenous analgesic activity, and its release can inhibit the onset of pain (22). In addition to its analgesic effects in the central nervous system, increased β-EP in peripheral tissues also influences peripheral and local analgesia.

The COX-2/mPGES-1/PGE2 pathway influences the progression of primary dysmenorrhea by promoting inflammatory responses and inducing oxidative stress (23, 24). β-EP is also closely associated with primary dysmenorrhea. This study aims to investigate the analgesic effects of low- frequency and high-frequency electroacupuncture pretreatment in a rat model of cold-coagulation type dysmenorrhea and to examine the impact of different frequencies of electroacupuncture on the COX-2/mPGES-1/PGE2 pathway-related indicators and β-EP in various tissues, thereby exploring the analgesic mechanisms of different frequencies of electroacupuncture in PD.

## Experimental materials

### Experimental animals and grouping

Forty three-month-old SPF-grade healthy female Sprague-Dawley (SD) rats, weighing (220 ± 20) g and sexually mature but unbred, were provided by SPF (Beijing) Biotechnology Co., Ltd., License No.: SYXK(Jing)2020-0013. During the experimental design, we tried to minimize the impact of individual differences by ensuring that the baseline body weights of the rats in each group were not statistically significantly different (*P* > 0.05) before the experiment officially began.

The animals were housed in an environment with a temperature of (20 ± 5) °C and humidity of (50 ± 5) %, fed standard laboratory chow, and given free access to water. Rats in the estrus phase were selected using a vaginal smear methylene blue staining method (25, 26).

After selection, we coded the rats and randomly assigned them to one of four groups using SPSS software: blank control group (control group), cold-coagulation type dysmenorrhea model group (model group), low-frequency electroacupuncture group (LF-EA group, 2/10 Hz), and high-frequency electroacupuncture group (HF-EA group, 20/100 Hz), with 10 rats in each group. Please see the spreadsheet titled SPSS-Randomized Rat Groups in the **Supplementary Materials** for more details. Rat writhing responses were scored by observers trained in the standardized writhing response scoring protocol but uninvolved in model induction or acupuncture treatment, with the observers blinded to group assignments. We set the sample size at 10 per group, slightly exceeding the lower end of the common range (6–8) for experimental dysmenorrhea studies involving rats (27–30). This decision was influenced by the constraints of resources and funding, while also balancing the need for sufficient statistical power.

### Main experimental reagents and instruments

Reagents: Benzoic Estradiol Injection (Shanghai Quanyu Biotechnology (Zhumadian) Animal Pharmaceutical Co., Ltd., Batch No.: 210106); Oxytocin Injection (Jiangxi Bolai Pharmacy Co., Ltd., Batch No.: Animal No. 140062778); HE Staining Kit (Beijing Zhongke Wanbang Biotechnology Co., Ltd., RY-0002); Paraffin (Leica 39601095); The Rat Cyclooxygenase-2 (COX-2) ELISA Kit (RGB & CHN, Lot: 20250418.60502R, manufactured in Finland); Rat Membrane-Bound Prostaglandin E Synthase-1 (mPGES-1) ELISA Kit (RGB & CHN, Lot: 20250418.60753R, manufactured in Finland); Rat Prostaglandin E2 (PGE2) ELISA Kit (RGB & CHN, Lot: 20250418.60069R, manufactured in Finland); Rat Prostaglandin F2α (PGF2α) ELISA Kit (RGB & CHN, Lot: 20250418.60469R, manufactured in Finland); Rat β-Endorphin (β-EP) ELISA Kit (RGB & CHN, Lot: 20250418.60235R, manufactured in Finland); COX-2 Antibody (Affinity, Catalog No. AF7003); mPGES-1 Antibody (Bioss, Catalog No. BS-1880R); Beta-Endorphin Antibody (Affinity, Catalog No. DF7154); Prostaglandin E Receptor EP2 Antibody (Affinity, Catalog No. DF4878).

Instruments: Centrifuge (Beijing Baiyang Medical Equipment Co., Ltd., BY-300C); Dehydrator (Wuhan Junjie Electronics Co., Ltd., JT-12S); Embedding Machine (Wuhan Junjie Electronics Co., Ltd., JB-P7); Paraffin Slicer (Leica RM2235, Germany); BX51 Microscope (Olympus Corporation, Japan); Beckmancoulter UniCel DxC 600 Synchron Fully Automated Biochemical Analyzer (Origin: USA); Electrophoresis Apparatus (Bio-Rad 1645070); Electroporator (Longfang Xingyu, LF-600S); Microplate Reader (Thermo Scientific AF1119500); Electronic Balance (BT223S, Beijing Sartorius Instruments & System Engineering Co., Ltd.); Ice Maker (SANYO, SIM-F123, Japan); Disposable Sterile Acupuncture Needles (0.16 × 13 mm and 0.18 × 25 mm, Beijing Zhongyan Taihe Medical Equipment Co., Ltd.); Hwato SDZ-IIB Electroacupuncture Instrument (Suzhou Medical Supplies Factory Co. Ltd).

### Model preparation

A cold-coagulation type dysmenorrhea rat model was established by combining a (0 ± 1)°C ice water bath with benzoic estradiol and oxytocin administration (29, 31). On days 1 and 10, each rat received an injection of 0.5 mg per rat. From days 2 to 9, each rat was subcutaneously injected with 0.2 mg per day. On day 11, the model group, LF-EA group, and HF-EA group received an intraperitoneal injection of 2 U oxytocin per rat. From day 1 to day 10 of model induction, rats in the model group, LF-EA group, and HF-EA group were subjected to cold stimulation by placing them in an ice water mixture maintained at (0 ± 1)°C within an environment at room temperature (20 ± 5)°C. The ice water bath level was adjusted to immerse the rats’ hind limbs and lower abdomen. This procedure was conducted once daily for 20 minutes each session. After 10 days of continuous cold stimulation, the model rats exhibited clear cold-coagulation symptoms and signs, including shivering, huddling with reduced movement, preference for stacking, purplish discoloration of claws and lips, and loose stools. The control group only received intraperitoneal and subcutaneous injections of 0.9% sodium chloride solution in equivalent milliliter volumes.

### Intervention methods

In designing our study, we carefully considered the optimal timing for EA intervention based on existing research findings, which indicate that pre-emptive acupuncture (including EA) is more effective than immediate acupuncture (including EA) (32, 33). Additionally, another study suggests that longer-duration or multiple-cycle treatments yield similar efficacy to shorter-duration or single-cycle treatments (25).

Electroacupuncture interventions for the LF-EA and HF-EA groups commenced on the 8th day of model induction (3 days in advance) and continued for 3 consecutive days. During the intervention period (days 8, 9, and 10), estradiol injections were still administered. Acupoint selection was based on “Common Acupoints and Locations for Experimental Animals Part 2: Rats” (34). For the Guanyuan(CV4) acupoint, needles were inserted vertically to a depth of 5 mm, followed by uniform lifting, thrusting, and twisting techniques without retaining the needles. Bilateral Ciliao (BL32) acupoints were inserted obliquely at a 45° angle to a depth of 10–15 mm (35), with uniform lifting, thrusting, and twisting techniques for 30 seconds. The Sanyinjiao(SP6) acupoint was inserted vertically to a depth of 5 mm, followed by uniform lifting, thrusting, and twisting techniques. Bilateral Ciliao acupoints were connected for electroacupuncture using dense-sparse waveforms, with frequencies set at 2/10 Hz for the LF-EA group and 20/100 Hz for the HF-EA group. The current intensity was adjusted to induce slight local muscle tremors without causing the rats to become agitated or struggle. Needles were retained for 20 minutes. The control group and model group were subjected to the same method of handling and fixation but did not receive any electroacupuncture treatment.

More detailed acupuncture procedures can be found in the **Supplementary Materials**.

### Collection of rat-related indicators

After the observation of the writhing response in the rats, they were weighed and anesthetized via intramuscular injection of 1% sodium pentobarbital (50 mg/kg) (36). Five milliliters of blood were collected from the abdominal aorta and allowed to stand at room temperature for 20 minutes. The blood was then centrifuged at 4°C and 3,200 r/min for 10 minutes. The supernatant (serum) was collected and stored in cryovials at −80°C for subsequent analyses. The rats were then euthanized, and the hypothalamus and spinal cord were rapidly dissected, first immersed in liquid nitrogen and subsequently transferred to a -80°C freezer for preservation. Following this, the uterus, ovaries (bilateral), liver, spleen, and kidneys (bilateral) were dissected and rinsed with 0.9% sodium chloride solution. The tissues were blotted dry with filter paper and weighed. For each group, the left uterus of each rat was fixed in 4% paraformaldehyde, while the right uterus was stored at -80°C for further use.

### Observation indicators and detection methods

Rats were weighed before and after treatment to observe changes in body weight. Writhing Response Observation: On the 11th day, after intraperitoneal injection of oxytocin, the writhing responses of rats in each group were observed and recorded for 20 minutes, documenting the writhing latency and score (37). Writhing latency is defined as the time from intraperitoneal injection of oxytocin to the onset of writhing responses. Writhing behaviors were classified into four grades. Grade 0: Normal posture (forepaws flat on the bottom of the container or exhibiting normal exploratory behavior); Grade 1: Body tilts to the left or right; Grade 2: Hind limbs extend, hind paws dorsiflexed, and body stretches with frequent pelvic lateral rotations; Grade 3: Abdominal muscle contractions accompanied by body and hind limb extension. Writhing score was calculated as follows: Writhing Score = (Number of Grade 0 × 0) + (Number of Grade 1 × 1) + (Number of Grade 2 × 2) + (Number of Grade 3 × 3).

Calculation of Organ Indices for Uterus, Ovaries (Bilateral), Liver, Spleen, and Kidneys (Bilateral).

Excess fat was removed from the uterus, ovaries (bilateral), liver, spleen, and kidneys (bilateral) of the rats. The surfaces of the tissues were rinsed with 0.9% sodium chloride solution, blotted dry with filter paper, and accurately weighed. Organ indices were calculated using the following formula: Organ Index = Organ Weight (mg)/Body Weight (g) (38–40).

HE Staining for Uterine Histopathology and Pathological Scoring: Uterine tissues fixed in 4% paraformaldehyde were processed for paraffin embedding, sectioned, deparaffinized, stained with hematoxylin and eosin (HE), dehydrated, and mounted on slides. Histological features were examined under an optical microscope, and morphological characteristics were captured via microscopy. Image acquisition was subsequently performed.

We measured the levels of COX-2, mPGES-1, PGE2, PGF2α, and β-EP in the serum, uterine tissue, hypothalamus, and spinal cord using the ELISA method, strictly following the protocols provided in the ELISA kit manuals.

Using Western blot analysis, we detected the protein expression levels of COX-2, mPGES-1, EP2, and β-EP in uterine and spinal cord tissues preserved at −80°C. We lysed tissue samples with lysis buffer and measured protein concentrations using the BCA Protein Assay Kit. Proteins were subjected to electrophoresis and transferred to PVDF membranes. The membranes were blocked at room temperature for 90 minutes and then incubated overnight at 4°C with primary antibodies diluted in 3% skim milk (COX-2: 1:1000, mPGES-1: 1:1000, EP2: 1:1000, β-EP: 1:1000). After being washed, membranes were incubated with HRP-conjugated secondary antibodies diluted in 1× TBST (1:20000) at room temperature on a shaker for 1 hour. GAPDH was used as the internal reference protein. We exposed the membranes using an automated chemiluminescence imaging system and analyzed the results using ImageJ software. The relative expression levels of target proteins were calculated as the ratio of the target protein to GAPDH.

### Statistical analysis

During the process of model induction with a (0 ± 1)°C ice-water bath, one rat in the HF-EA group unfortunately drowned and died. Consequently, data were collected from the remaining 9 rats in the HF-EA group, while the control, model, and LF-EA groups each retained their full cohort of 10 rats.

Prior to statistical analysis, outliers were identified and excluded to ensure robustness. Specifically, the HF-EA group excluded one outlier, retaining 8 rats; the control, model, and LF-EA groups excluded two outliers per group, retaining 8 rats per group. Final analysis included data from 8 rats per group to maintain methodological consistency.

We conducted comprehensive tests for normality (*P* > 0.05) and homogeneity of variance (using Levene’s Test) for all datasets. All data were expressed as mean ± standard deviation ( $\bar{x}$ **± s**). Statistical analyses were performed using SAS 9.4 software. Our statistical methods to test the normality and homogeneity of variance of the data are outlined as follows:

1. One-Way ANOVA with Levene’s Test for Homogeneity of Variance:

Our experimental design comprises four groups: control group, model group, LF-EA group and HF-EA group. For each observed indicator, four corresponding sets of data were generated.

For datasets that conform to a normal distribution, we first perform Levene’s Test to assess homogeneity of variance. If the variances are homogeneous (*P* > 0.05) and the groups exhibit significant differences (P < 0.05), we proceed with LSD (Least Significant Difference) *post-hoc* tests for pairwise comparisons among the groups.

2. Welch’s F-test for Datasets with Unequal Variances:

If the datasets conform to a normal distribution but exhibit unequal variances, we employ Welch’s F-test. Should the groups demonstrate significant differences (*P* < 0.05), we again use LSD for pairwise comparisons.

3. Kruskal-Wallis Test for Non-Normal Datasets:

For datasets that do not conform to a normal distribution, we conduct the Kruskal-Wallis Test as a non-parametric alternative. If the overall test indicates significant differences among the groups (*P* < 0.05), we proceed with Wilcoxon rank-sum tests for pairwise comparisons.

4. Comparisons using non-parametric (Wilcoxon) tests:

For scenarios involving only two groups, if the data are normally distributed, we perform a T-Test. If the data are not normally distributed, we rely on the results of the Wilcoxon rank-sum test (two-tailed).

## Results

### Screening of rat estrus cycle

**Figure 1:**
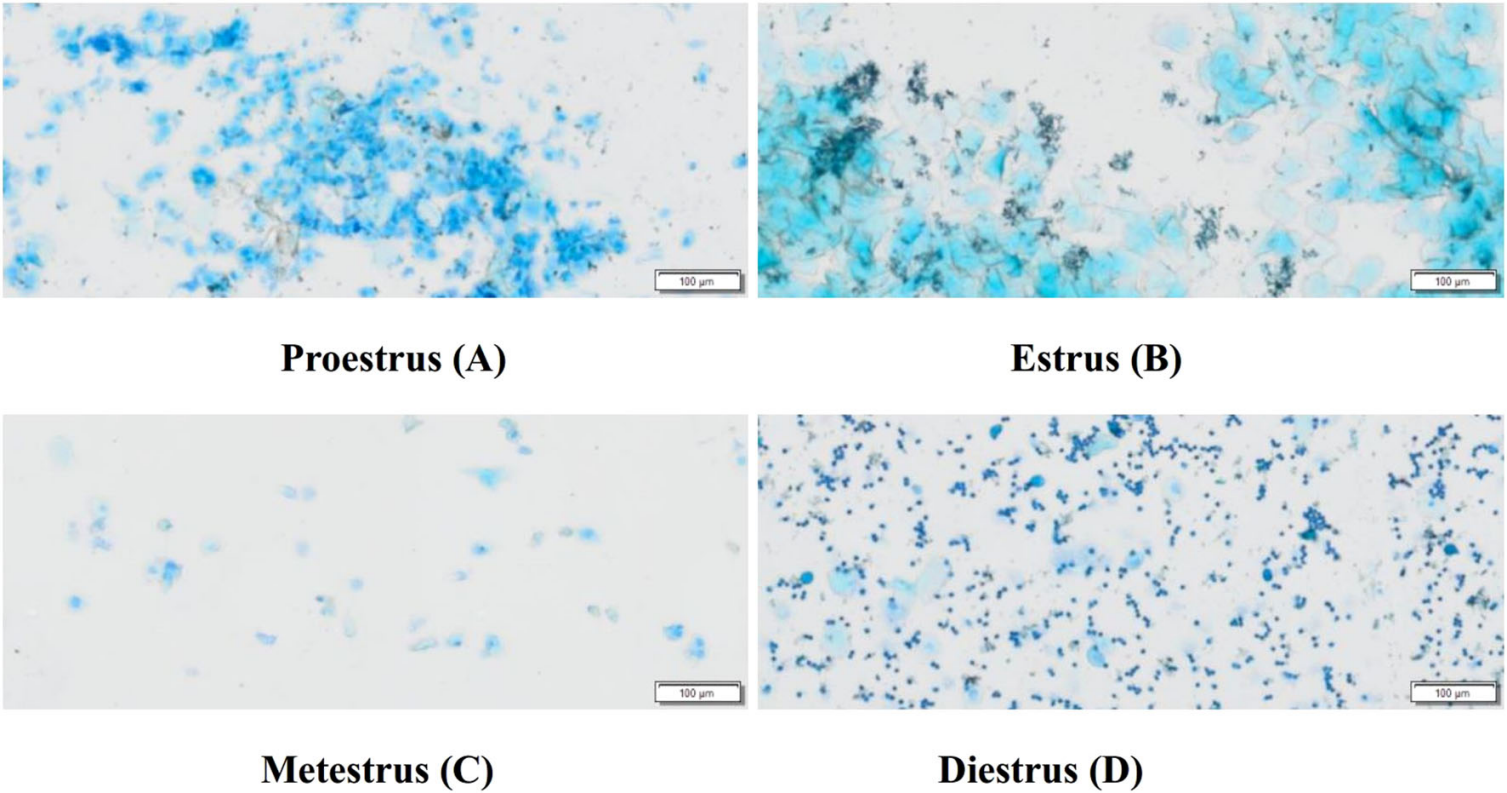
Typical vaginal smears of rats at different stages of the estrus cycle (Scale bar = 100 μm). Proestrus **(A)**, Estrus **(B)**, Metestrus **(C)**, Diestrus **(D)**.

The complete estrus cycle of rats lasts 4 to 5 days and is divided into four phases. Proestrus (A): Vaginal smears predominantly contain nucleated epithelial cells, accompanied by a few keratinized epithelial cells. The nuclei of epithelial cells are lightly stained, enlarged, and show signs of dissolution. Estrus (B) Smears are covered with large areas of fallen-leaf-like keratinized epithelial cells. Metestrus (C): Smears consistently show nucleated epithelial cells, keratinized epithelial cells, and leukocytes. Diestrus (D): Smears contain a large number of leukocytes and a few nucleated epithelial cells (25, 26). Refer to **Figure 1**.

**Figure 2:**
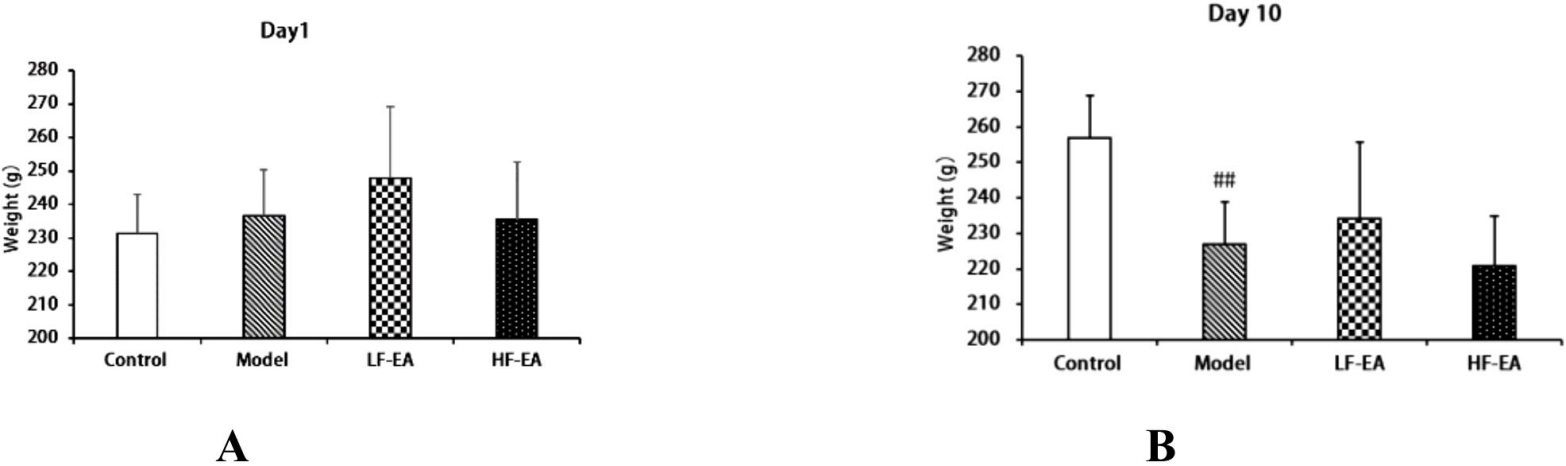
Changes in body weight of rats. **(A)** Baseline body weight before treatment. **(B)** Body weight after treatment. Data are expressed as mean ± SD. Control (n=10), model (n=10), LF-EA (n=10), HF-EA (n=9). Statistical significance: ##P < 0.01 vs. control.

Before model induction, there were no significant differences in the baseline body weights among the groups (*P* > 0.05), as shown in **Figure 2A**. On the 10th day of model induction, the body weight of the model group significantly decreased compared to the control group (*P* < 0.01). There were no statistically significant differences in body weight after treatment between the LF-EA group and the HF-EA group (*P* > 0.05), as illustrated in **Figure 2B**.

### Observation of writhing responses in each group of rats

**Figure 3:**
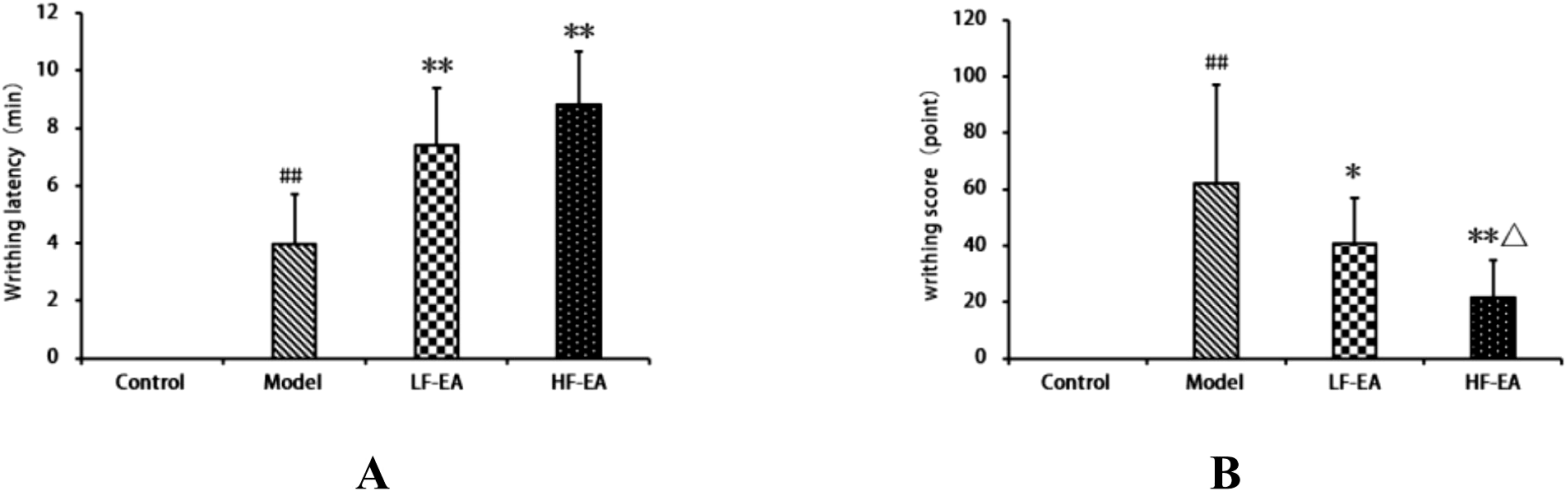
Observation of writhing responses in each group of rats. **(A)** Writhing latency (time from oxytocin injection to onset of writhing). **(B)** Writhing score (graded pain intensity based on behavioral criteria). Data are expressed as mean ± SD. Control (n=10), model (n=10), LF-EA (n=10), HF-EA (n=9). Statistical significance: ##P < 0.01 vs. control; *P < 0.05, **P < 0.01 vs. model; △P < 0.05 vs. LF-EA.

The control group exhibited no writhing responses. Compared to the control group, the writhing latency of the model group significantly increased (*P* < 0.01). Compared to the model group, both the LF-EA group and the HF-EA group showed significant increases in writhing latency (*P* < 0.01), as shown in **Figure 3A**. Compared to the control group, the writhing score of the model group significantly increased (*P* < 0.01). Compared to the model group, the writhing score increased in the LF-EA group (*P* < 0.05) and significantly increased in the HF-EA group (*P* < 0.01). Compared to the LF-EA group, the writhing score decreased in the HF-EA group (*P* < 0.05), as depicted in **Figure 3B**. The absence of writhing responses in the control group and the significant increases in writhing latency and writhing scores in the model group indicate the successful establishment of the PD model.

### Comparison of uterine morphology in each group of rats

**Figure 4:**
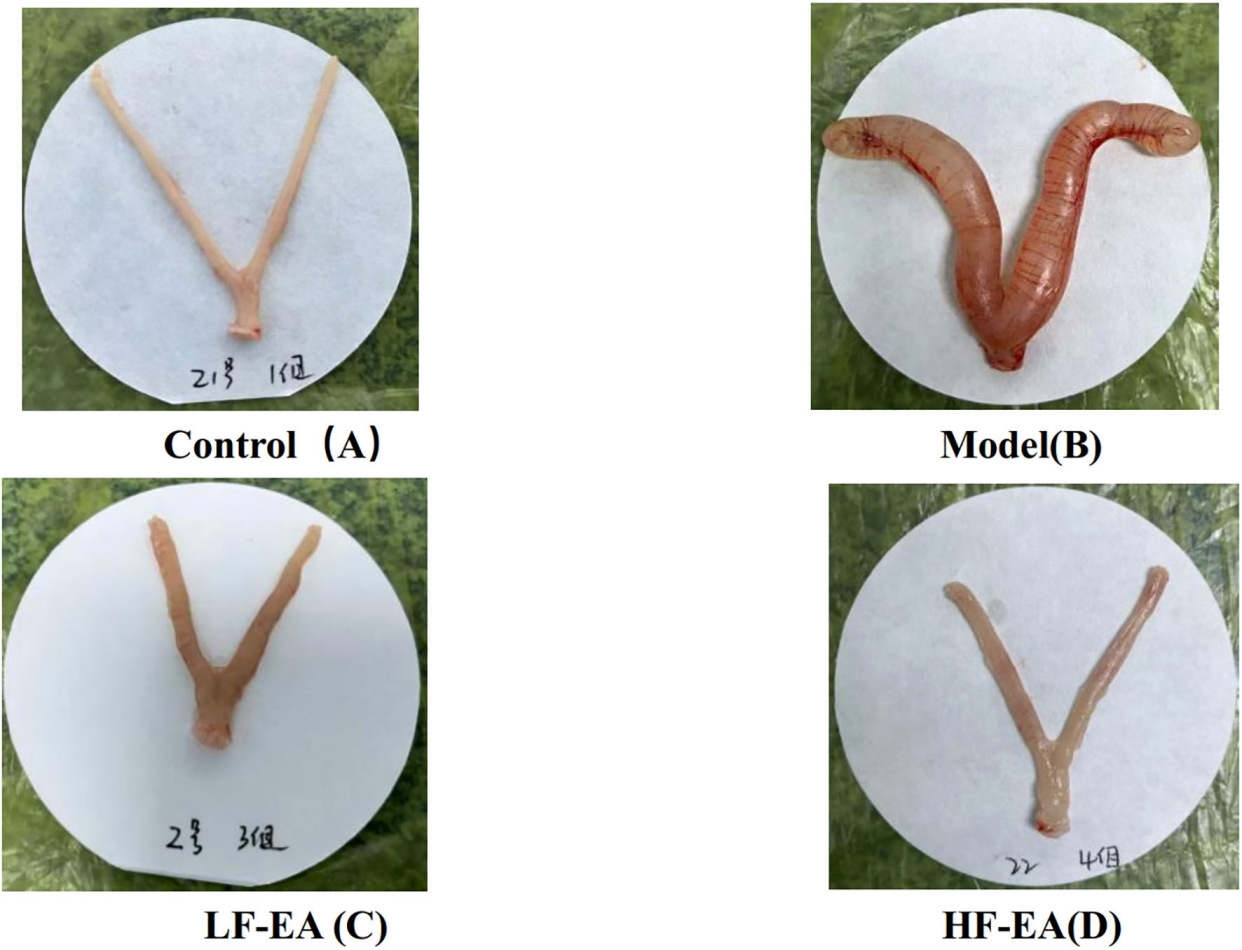
Comparison of uterine morphology in each group of rats (control, n=10; model, n=10, LF-EA, n=10, HF-EA, n=9). Control **(A)**, Model **(B)**, LF-EA **(C)**, HF-EA **(D)**.

Gross observation revealed that the uterus of rats in the control group appeared normal (**Figure 4A**). In contrast, the model group exhibited obvious uterine congestion and edema, with severe inflammatory edema (**Figure 4B**). The LF-EA group and the HF-EA group showed a reduction in uterine inflammatory edema, as illustrated in **Figures 4C, D**.

### Comparison of organ indices among each group of rats

**Figure 5:**
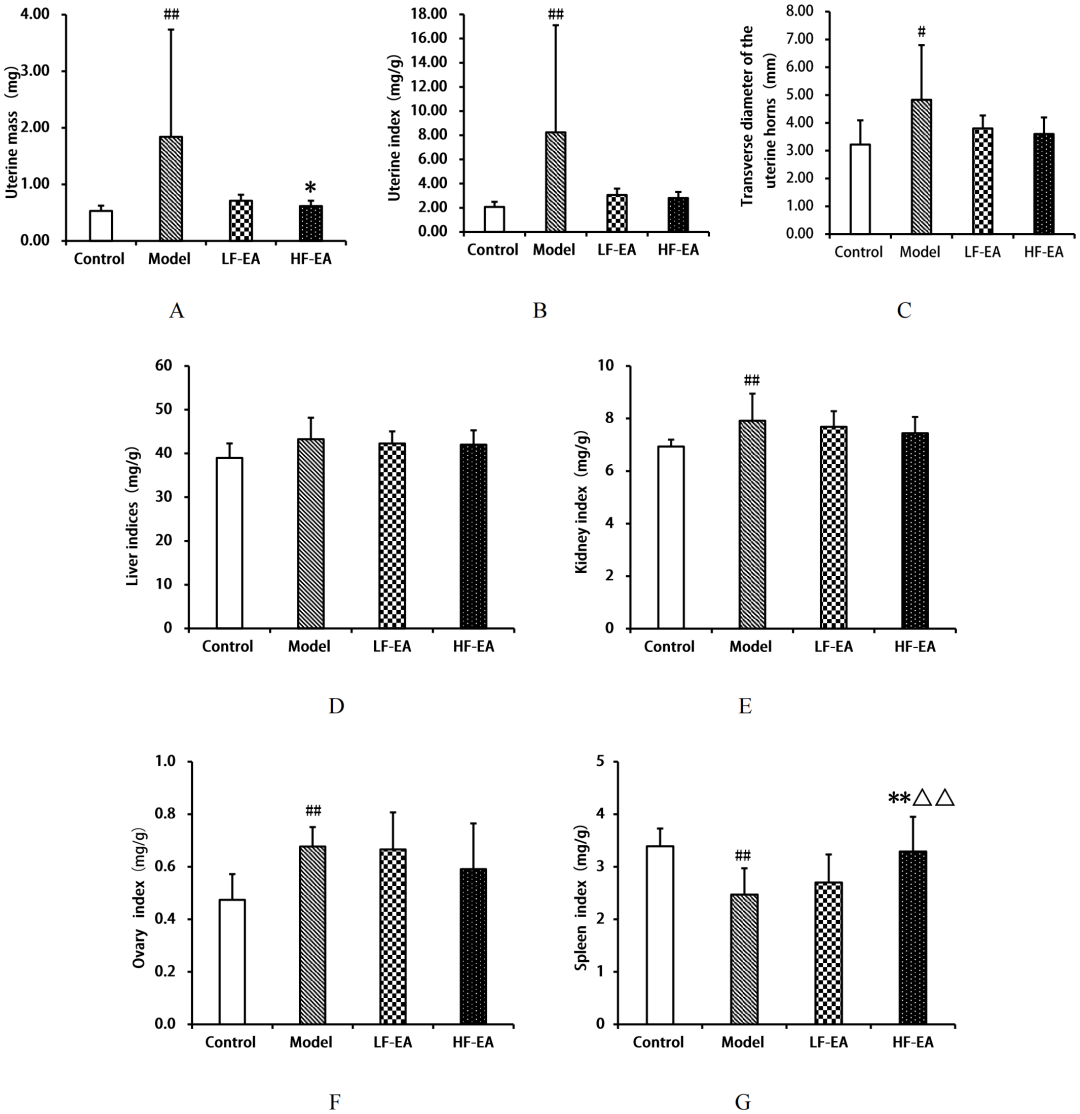
Comparison of organ indices. **(A)** Uterine mass. **(B)** Uterine index. **(C)** Transverse diameter of uterine horns. **(D)** Liver index. **(E)** Kidney index. **(F)** Ovary index. **(G)** Spleen index. Data are expressed as mean ± SD. Control (n=10), model (n=10), LF-EA (n=10), HF-EA (n=9). Statistical significance: #P < 0.05, ##P < 0.01 vs. control; *P < 0.05,**P < 0.01 vs. model; △△P < 0.01 vs. LF-EA.

Compared to the control group, the model group exhibited a significant increase in uterine mass(A), uterine index(B), kidney index(E), ovary index(F), (*P* < 0.01), and transverse diameter of the uterine horns(C) (*P* < 0.05), and a significant decrease in spleen index(G) (*P* < 0.01). Compared to the model group, the LF-EA group showed a decreasing trend in uterine mass (*P* > 0.05), while the HF-EA group demonstrated reductions in uterine mass (*P* < 0.05), both the LF-EA group and the HF-EA group showed a trend towards reduced uterine index, transverse diameter of the uterine horns, kidney index, and ovary index (*P* > 0.05). Additionally, the LF-EA group exhibited an increasing trend in spleen index (*P* > 0.05), whereas the HF-EA group showed a significant increase in spleen index compared to the LF-EA group (*P* < 0.01). Furthermore, when comparing the HF-EA group to the LF-EA group, the HF-EA group had a significantly higher spleen index (*P* < 0.01), while there were no statistically significant differences in other indices between the HF-EA and LF-EA groups (*P* > 0.05). There were no significant differences in liver indices among the groups (*P* > 0.05) (D). Refer to **Figure 5**.

### Comparison of uterine tissue histopathology and pathological scoring by the staining among each group of rats

**Figure 6:**
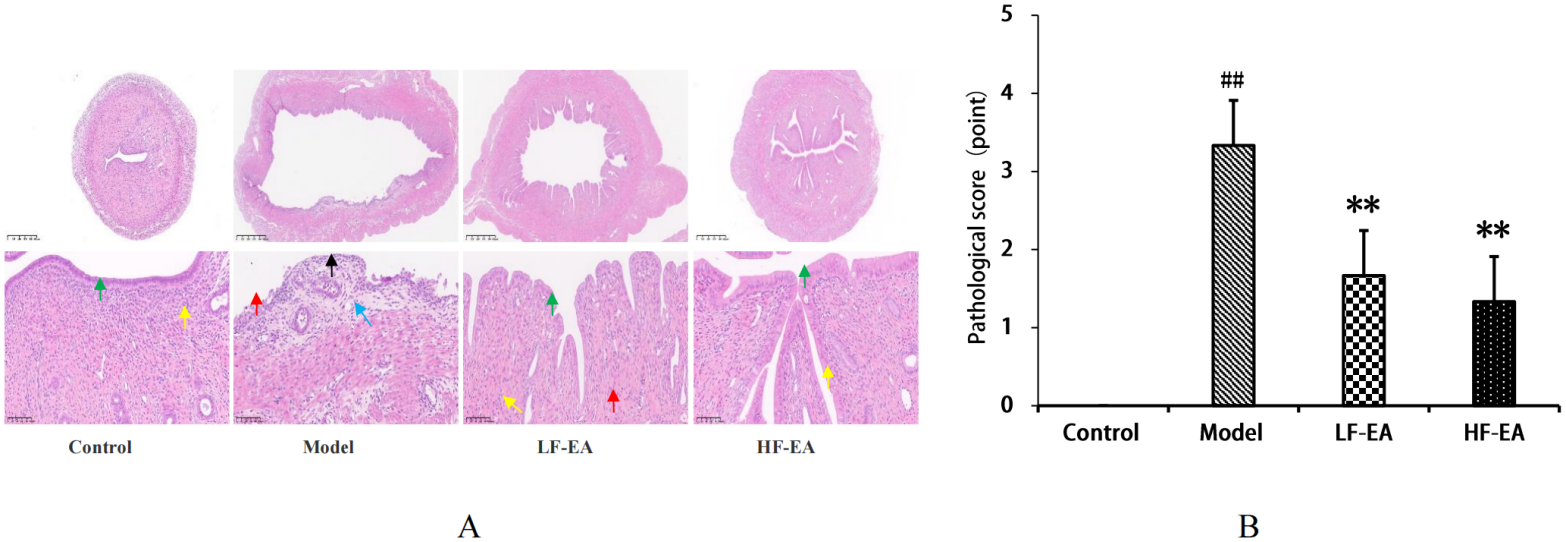
Uterine histopathology and pathological scores. **(A)** Representative HE-stained images. **(B)** Quantitative pathological scores. Data are expressed as mean ± SD. (n=3, per group). Statistical significance: ##P < 0.01 vs. control; **P < 0.01 vs. model.

In the control group, the uterine structure was normal, with a regular uterine cavity shape, orderly arrangement of epithelial cells, clear cytoplasmic outlines, and no obvious degeneration (green arrows). The lamina propria was thickened, with a higher number of uterine endometrial glands (yellow arrows), and no significant infiltration of inflammatory cells in the tissues. In contrast, the model group exhibited severe uterine damage, including significant expansion of the uterine cavity, erosion and detachment of epithelial cells in some areas, exposure of the lamina propria (black arrows), thinning of the lamina propria, massive infiltration of inflammatory cells (red arrows), fibrosis (blue arrows), and a significant reduction in the number of uterine endometrial glands. The LF-EA group showed some improvement in the degree of damage compared to the model group, with contraction of the uterine cavity, intact epithelium without detachment, partial vacuolar degeneration of epithelial cells, loose cytoplasm (green arrows), thickened lamina propria, an increased number of uterine endometrial glands (yellow arrows), and slight infiltration of inflammatory cells (red arrows). The HF-EA group exhibited further alleviation of damage, with a more regular uterine cavity, intact epithelium without obvious degeneration or detachment (green arrows), thicker lamina propria, and an increased number of uterine endometrial glands (yellow arrows). Pathological scoring was based on microscopic evaluation, with cumulative scores assigned according to standardized criteria (41). No significant pathological changes were observed in the uterine tissues of the control group. Refer to **Figure 6A**. Compared to the control group, the model group showed a significant increase in pathological scores (*P* < 0.01). Compared to the model group, the LF-EA group and the HF-EA group showed a significant decrease in pathological scores (*P* < 0.01). There was no statistically significant difference in pathological scores between the LF-EA and HF-EA groups (*P* > 0.05). Refer to **Figure 6B**.

### Comparison of COX-2, mPGES-1, PGE2, PGF2α, β-EP levels, and PGF2α/PGE2 ratio in serum among each group of rats

**Figure 7:**
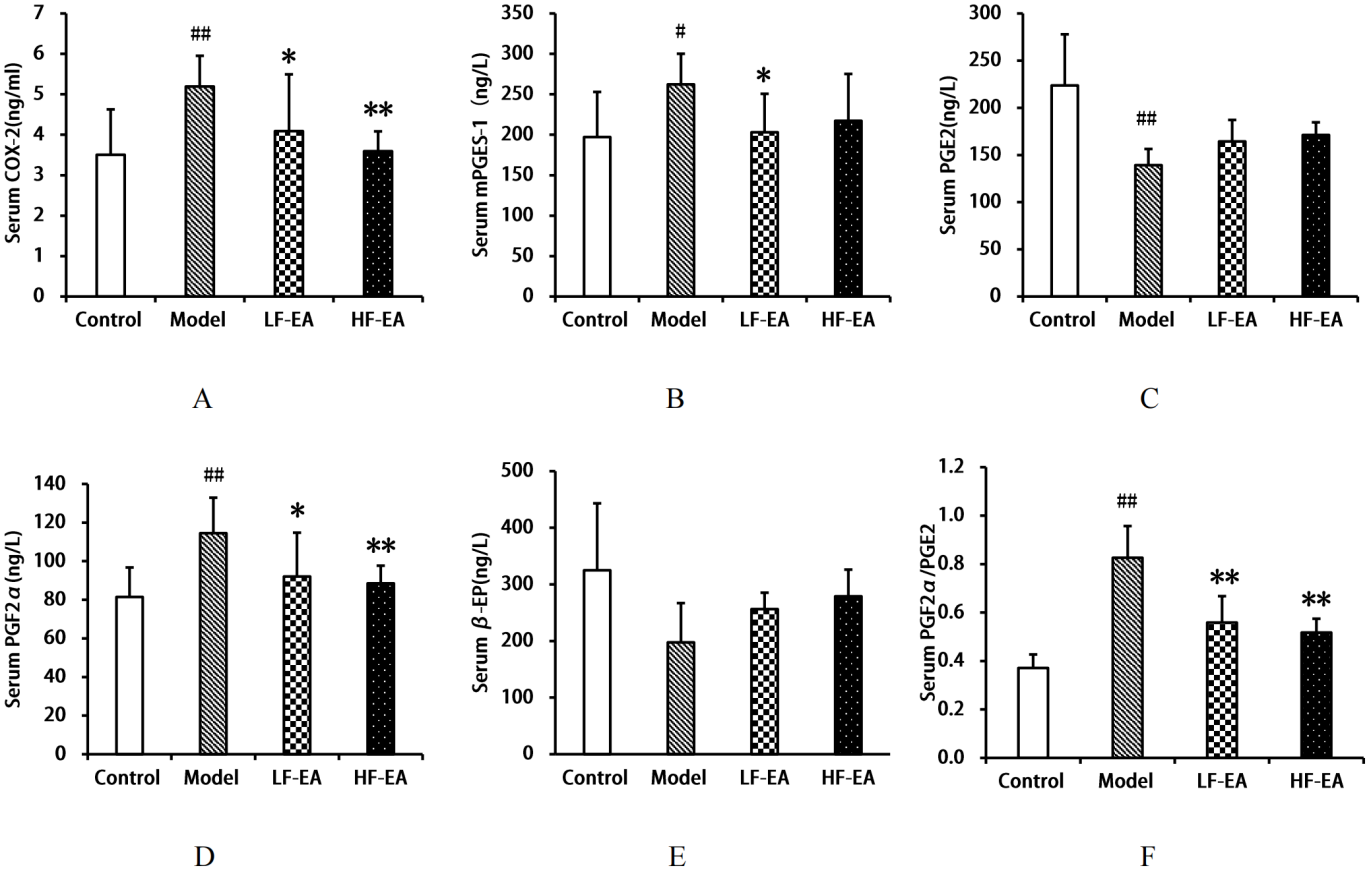
Serum levels of COX-2, mPGES-1, PGE2, PGF2α, β-EP, and PGF2α/PGE2 ratio. **(A)** COX-2. **(B)** mPGES-1. **(C)** PGE2. **(D)** PGF2α. **(E)** β-EP. **(F)** PGF2α/PGE2 ratio. Data are expressed as mean ± SD. Control (n=8), model (n=8), LF-EA (n=8), HF-EA (n=8). Statistical significance: #P < 0.05, ##P < 0.01 vs. control; *P < 0.05, *P < 0.01 vs. model.

Compared to the control group, the model group exhibited significant increases in serum COX-2(A) and PGF2αlevels (D), as well as the PGF2α/PGE2 ratio(F) (*P* < 0.01), and an increase in mPGES-1 levels(B) (*P* < 0.05), while PGE2 levels(C) significantly decreased (*P* < 0.01). Compared to the model group, the LF-EA group showed reductions in COX-2, mPGES-1, and PGF2α levels (*P* < 0.05), and a significant decrease in the PGF2α/PGE2 ratio (*P* < 0.01), with an increasing trend in PGE2 levels (*P* > 0.05). The HF-EA group demonstrated significant reductions in COX-2, PGF2α levels, and the PGF2α/PGE2 ratio (*P* < 0.01), with an increasing trend in mPGES-1 and PGE2 levels (*P* > 0.05). There were no statistically significant differences in β-EP levels (E) among the groups (*P* > 0.05). Comparing the LF-EA and HF-EA groups, there were no statistically significant differences in serum COX-2, mPGES-1, PGE2, PGF2α, β-EP levels, and the PGF2α/PGE2 ratio (*P* > 0.05). Refer to **Figure 7**.

### Comparison of COX-2, mPGES-1, PGE2, PGF2α, β-EP levels, and PGF2α/PGE2 ratio in the uterus among each group of rats

**Figure 8:**
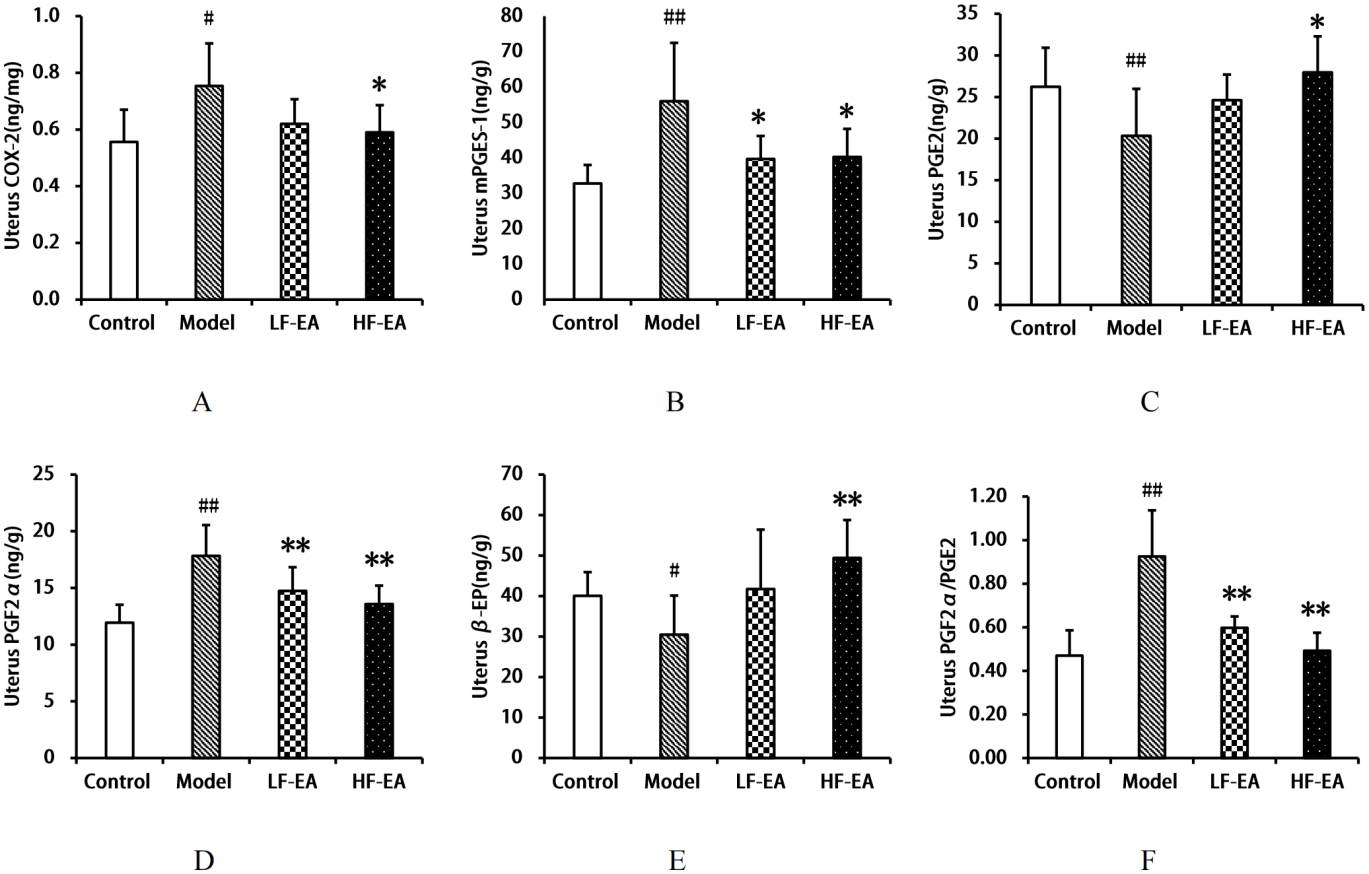
Uterine levels of COX-2, mPGES-1, PGE2, PGF2α, β-EP, and PGF2α/PGE2 ratio. **(A)** COX-2. **(B)** mPGES-1. **(C)** PGE2. **(D)** PGF2α. **(E)** β-EP. **(F)** PGF2α/PGE2 ratio. Data are expressed as mean ± SD. Control (n=8), model (n=8), LF-EA (n=8), HF-EA (n=8). Statistical significance: #*P* < 0.05, ##*P* < 0.01 vs. control; *P < 0.05, **P < 0.01 vs. model.

Compared to the control group, the model group exhibited an increase in uterine COX-2 levels(A) (*P* < 0.05), significant increases in mPGES-1(B) and PGF2α(D) levels, and the PGF2α/PGE2 ratio(F) (*P* < 0.01), a significant decrease in PGE2 levels (C) (*P* < 0.01), and a decrease in β-EP levels (E) (*P* < 0.05). Compared to the model group, the LF-EA group showed a trend towards reduced COX-2 levels (*P* > 0.05), a decrease in mPGES-1 levels (*P* < 0.05), significant reductions in PGF2α levels and the PGF2α/PGE2 ratio (*P* < 0.01), and an increasing trend in PGE2 and β-EP levels (*P* > 0.05). The HF-EA group demonstrated significant reductions in COX-2 and mPGES-1 levels (*P* < 0.05), significant reductions in PGF2α levels and the PGF2α/PGE2 ratio (*P* < 0.01), an increase in PGE2 levels (*P* < 0.05), and a significant increase in β-EP levels (*P* < 0.01). Comparing the LF-EA and HF-EA groups, there were no statistically significant differences in uterine COX-2, mPGES-1, PGE2, PGF2α, β-EP levels, and the PGF2α/PGE2 ratio (*P* > 0.05). Refer to **Figure 8**.

### Comparison of COX-2, mPGES-1, PGE2, PGF2α, β-EP levels, and PGF2α/PGE2 ratio in the hypothalamus among each group of rats

**Figure 9:**
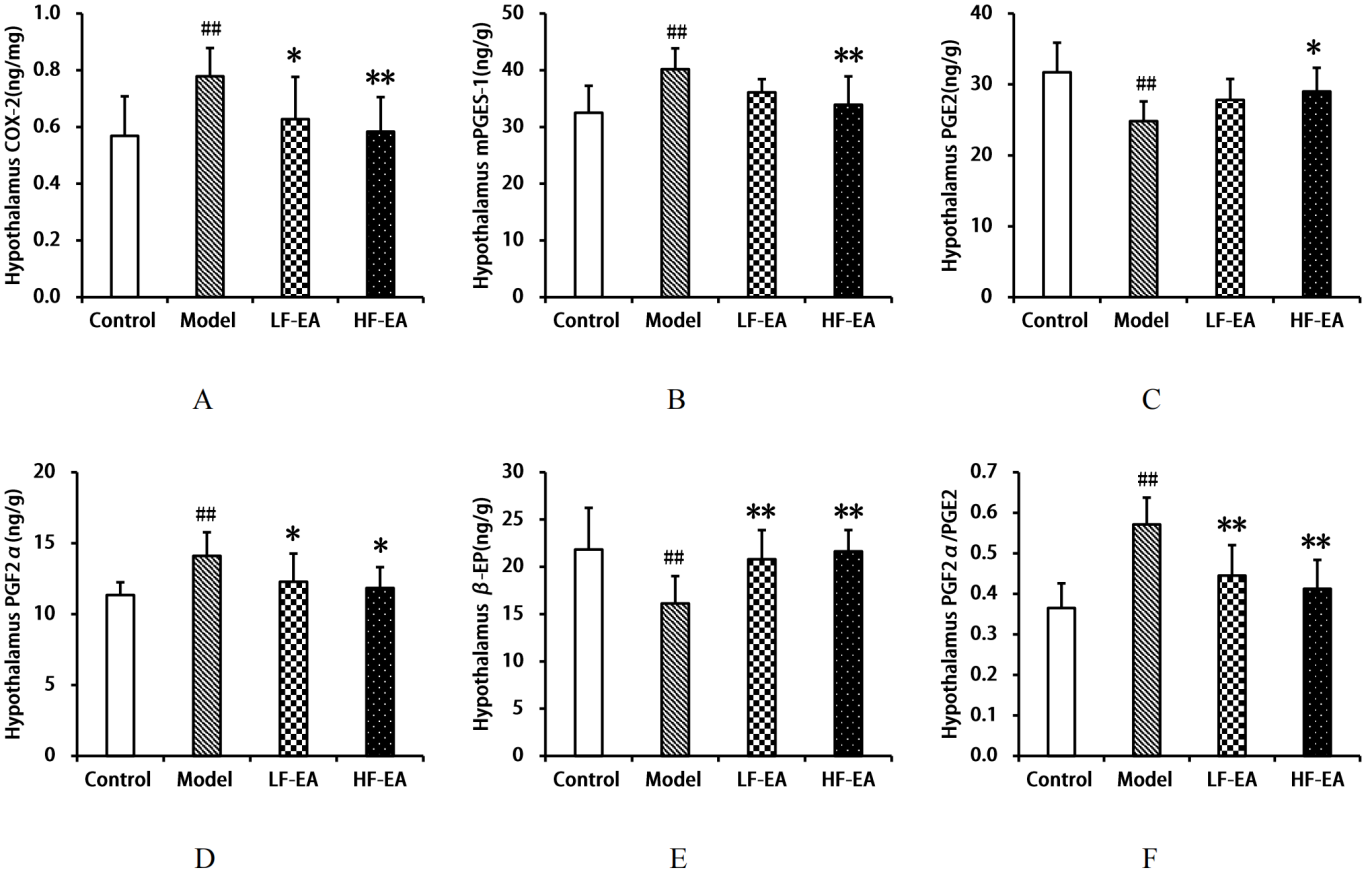
Hypothalamic levels of COX-2, mPGES-1, PGE2, PGF2α, β-EP, and PGF2α/PGE2 ratio. **(A)** COX-2. **(B)** mPGES-1. **(C)** PGE2. **(D)** PGF2α. **(E)** β-EP. **(F) **PGF2α/PGE2 ratio. Data are expressed as mean ± SD. Control (n=8), model (n=8), LF-EA (n=8), HF-EA (n=8). Statistical significance: ##P < 0.01 vs. control; *P < 0.05, **P < 0.01 vs. model.

Compared to the control group, the model group exhibited significant increases in hypothalamic COX-2(A), mPGES-1(B), PGF2α levels(D), and the PGF2α/PGE2 ratio(F) (*P* < 0.01), and significant decreases in PGE2(C) and β-EP levels(E) (*P* < 0.01). Compared to the model group, the LF-EA group showed reductions in COX-2 and PGF2α levels (*P* < 0.05), mPGES-1 levels with a trend towards decrease (*P* > 0.05), a significant decrease in the PGF2α/PGE2 ratio (*P* < 0.01), a trend towards increased PGE2 levels (*P* > 0.05), and a significant increase in β-EP levels (*P* < 0.01). The HF-EA group demonstrated significant reductions in COX-2, mPGES-1 levels, and the PGF2α/PGE2 ratio (*P* < 0.01), a decrease in PGF2α levels (*P* < 0.05), and an increase in PGE2 (*P* < 0.05) and a significant increase in β-EP levels (*P* < 0.01). There were no statistically significant differences between the LF-EA and HF-EA groups in hypothalamic COX-2, mPGES-1, PGE2, PGF2α, β-EP levels, and the PGF2α/PGE2 ratio (*P* > 0.05). Refer to **Figure 9**.

### Comparison of COX-2, mPGES-1, PGE2, PGF2α, β-EP levels, and PGF2α/PGE2 ratio in the spinal cord among each group of rats

**Figure 10:**
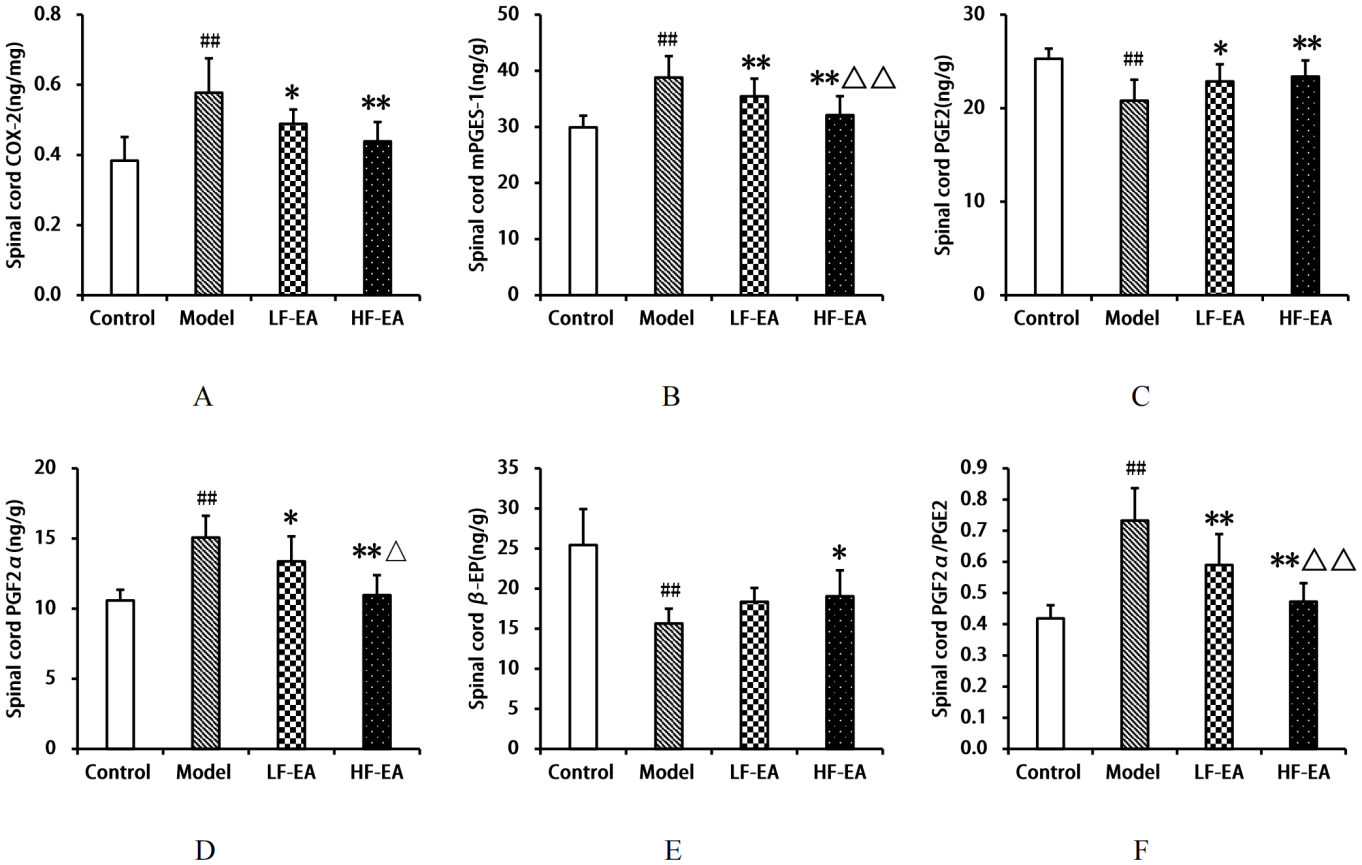
Spinal cord levels of COX-2, mPGES-1, PGE2, PGF2α, β-EP, and PGF2α/PGE2 ratio. **(A)** COX-2. **(B)** mPGES-1. **(C)** PGE2. **(D)** PGF2α. **(E)** β-EP. **(F)** PGF2α/PGE2 ratio. Data are expressed as mean ± SD. Control (n=8), model (n=8), LF-EA (n=8), HF-EA (n=8). Statistical significance: ##*P* < 0.01 vs. control; *P < 0.05, **P < 0.01 vs. model; △*P* < 0.05, △△*P* < 0.01 vs. LF-EA.

Compared to the control group, the model group exhibited significant increases in spinal COX-2(A), mPGES-1(B), PGF2α levels(D), and the PGF2α/PGE2 ratio(F) (*P* < 0.01), and significant decreases in PGE2(C) and β-EP levels(E) (*P* < 0.01). Compared to the model group, the LF-EA group showed decreases in COX-2 and PGF2α levels (*P* < 0.05), significant decreases in mPGES-1 levels and the PGF2α/PGE2 ratio (*P* < 0.01), an increase in PGE2 levels (*P* < 0.05), and a trend towards increased β-EP levels (*P* > 0.05). The HF-EA group demonstrated significant reductions in COX-2, mPGES-1, PGF2α levels, and the PGF2α/PGE2 ratio (*P* < 0.01), a significant increase in PGE2 levels (*P* < 0.01), and an increase in β-EP levels (*P* < 0.05). Compared to the LF-EA group, the HF-EA group showed significant reductions in mPGES-1 levels and the PGF2α/PGE2 ratio (*P* < 0.01), and a decrease in PGF2α levels (*P* < 0.05). Refer to **Figure 10**.

### Expression levels of COX-2, mPGES-1, EP2, and β-EP proteins in uterine tissue among each group of rats

**Figure 11:**
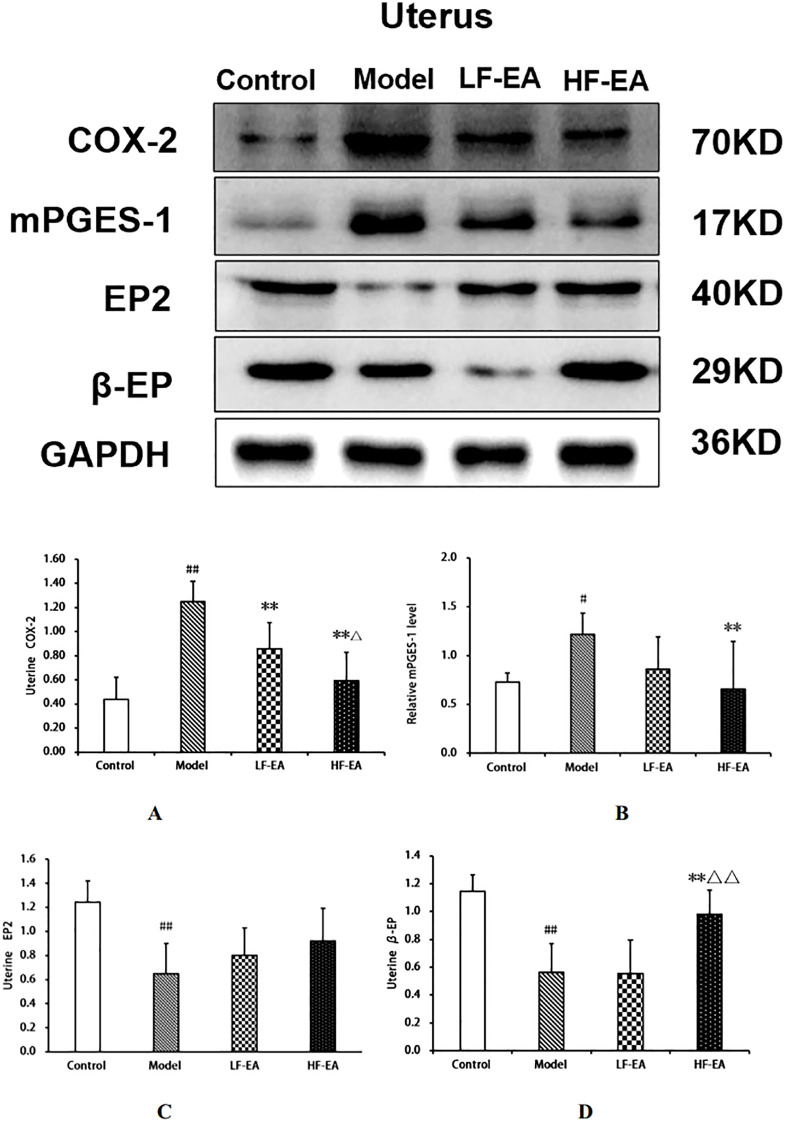
Uterine tissue protein expression of COX-2, mPGES-1, EP2, and β-EP. **(A)** COX-2. **(B)** mPGES-1. **(C)** EP2. **(D)** β-EP. Data are expressed as mean ± SD. Control (n=6), model (n=6), LF-EA (n=6), HF-EA (n=6). Statistical significance: #*P* < 0.05, ##*P* < 0.01 vs. control; **P < 0.01 vs. model; △*P* < 0.05, △△*P* < 0.01 vs. LF-EA.

Compared to the control group, the model group showed a significant increase in uterine COX-2 protein expression(A) (*P* < 0.01), an increase in mPGES-1 protein expression(B) (*P* < 0.05), and significant decreases in EP2 (C)and β-EP protein expressions (D) (*P* < 0.01). Compared to the model group, the LF-EA group exhibited a significant decrease in COX-2 protein expression (*P* < 0.01), a trend towards decreased mPGES-1 and β-EP protein expressions (*P* > 0.05), and a trend towards increased EP2 protein expression (*P* > 0.05). The HF-EA group demonstrated significant decreases in COX-2 and mPGES-1 protein expressions (*P* < 0.01), a trend towards increased EP2 protein expression (*P* > 0.05), and a significant increase in β-EP protein expression (*P* < 0.01). Compared to the LF-EA group, the HF-EA group showed a decrease in COX-2 protein expression (*P* < 0.05) and a significant increase in β-EP protein expression (*P* < 0.01). Refer to **Figure 11**.

### Expression levels of COX-2, mPGES-1, EP2, and β-EP proteins in the spinal cord among each group of rats

**Figure 12:**
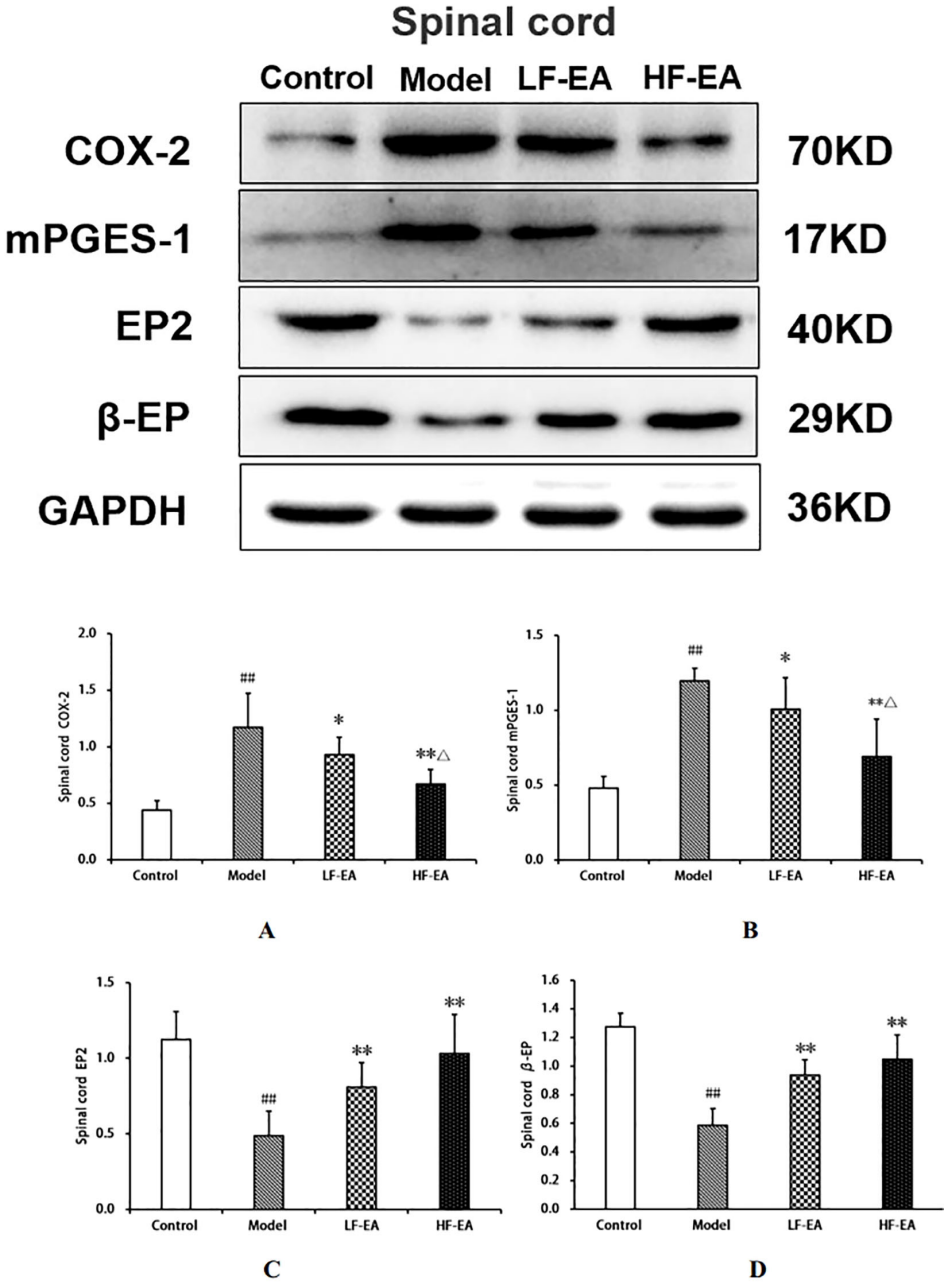
Uterine tissue protein expression of COX-2, mPGES-1, EP2, and β-EP. **(A)** COX-2. **(B)** mPGES-1. **(C)** EP2. **(D)** β-EP. Data are expressed as mean ± SD. Control (n=6), model (n=6), LF-EA (n=6), HF-EA (n=6). Statistical significance: #*P* < 0.05, ##*P* < 0.01 vs. control; **P < 0.01 vs. model; △*P* < 0.05, △△*P* < 0.01 vs. LF-EA.

Compared to the control group, the model group exhibited significant increases in spinal COX-2 (A)and mPGES-1 protein expressions (B)(*P* < 0.01), and significant decreases in EP2 (C)and β-EP protein expressions (D)(*P* < 0.01). Compared to the model group, the LF-EA group showed decreases in COX-2 and mPGES-1 protein expressions (*P* < 0.05), significant increases in EP2 and β-EP protein expressions (*P* < 0.01). The HF-EA group demonstrated significant decreases in COX-2 and mPGES-1 protein expressions (*P* < 0.01), and significant increases in EP2 and β-EP protein expressions (*P* < 0.01). Compared to the LF-EA group, the HF-EA group exhibited significant decreases in COX-2 and mPGES-1 protein expressions (*P* < 0.05). Refer to **Figure 12**.

## Discussion

Dysmenorrhea is a common clinical condition, also known as “menstrual abdominal pain”, which negatively impacts women’s quality of life (4). The most prevalent clinical subtype of Primary Dysmenorrhea (PD) is the old coagulation syndrome-type dysmenorrhea (42, 43). Therefore, this study selected a well-established rat model of cold-coagulation type dysmenorrhea (44), employing a combination of a (0 ± 1) °C ice water bath with benzoic estradiol and oxytocin to induce the model. The ice water bath aligns with the traditional Chinese medicine (TCM) concepts of “cold” and “coagulation”, thereby simulating the clinical manifestation of cold coagulation syndrome-type PD (45, 46). Estradiol increases uterine sensitivity, while oxytocin induces spastic uterine contractions that produce pain, thereby mimicking dysmenorrhea symptoms (47). The etiology and pathogenesis of PD can be summarized as “pain due to deficiency” or “pain due to obstruction”. The optimal timing for acupuncture treatment is 3–7 days before menstruation, allowing acupuncture to balance yin and yang, regulate deficiency and excess, soothe the spirit, adjust the patient’s emotional state, clear obstructions, and nourish deficiency, thereby achieving analgesic effects (48). Consequently, this study initiated acupuncture three days before menstruation. The Sanyinjiao acupoint is an empirically effective point for treating PD (49, 50), and the Ciliao acupoint is also commonly used for PD treatment (51). Given that this study focused on the cold-coagulation stagnation subtype, the Guanyuan acupoint, belonging to the Ren meridian, connects with the Chong meridian and is directly linked to the Bao Gong (uterus), providing functions such as warming the Bao Gong and tonifying primary yang. The combination of these three acupoints can regulate the Ren and Chong meridians, warm and smooth the Bao meridian, invigorate blood circulation, replenish blood, regulate menstruation, and alleviate pain.

The estrus cycle of rats consists of four phases: proestrus, estrus, metestrus, and diestrus. The functional state of the uterus, hormone levels, and behaviors of rats fluctuate with the estrus cycle, with diestrus being a period of relatively stable hormone levels (52). To enhance experimental accuracy, this study confirmed that all rats were in the same phase of the estrus cycle using the vaginal smear method before establishing the PD model. There were no significant differences in the baseline body weights of rats across groups before model induction. On the 10th day of model induction, high-frequency electroacupuncture was found to reduce the body weight of PD rats. In this study, the control group showed no writhing responses, whereas the model group exhibited significant writhing responses, indicated by markedly increased writhing latency and writhing scores, confirming the successful establishment of the PD model. Electroacupuncture was able to extend the writhing latency and increase writhing scores in dysmenorrheic rats, with high-frequency electroacupuncture proving superior to low-frequency electroacupuncture, suggesting that high-frequency electroacupuncture can alleviate dysmenorrhea symptoms. Gross observations revealed that the uterine tissues of rats in the control group appeared normal, while those in the model group showed obvious congestion and severe inflammatory edema. Both the LF-EA and HF-EA groups exhibited reduced inflammatory edema in uterine tissues, indicating that electroacupuncture effectively alleviates uterine inflammatory edema. Comparative analysis of uterine tissue histopathology and pathological scores through HE staining demonstrated that the HF-EA group had reduced uterine tissue damage and lower pathological scores compared to the LF-EA group, indicating that high-frequency electroacupuncture can decrease the extent of uterine tissue damage and improve the pathological state of the uterus.

PD is a prevalent gynecological disorder characterized by visceral pain, and its development is closely related to inflammatory and immune factors (53–55). Regarding organ indices, this study found that electroacupuncture can reduce uterine mass, uterine index, transverse diameter of the uterine horns, kidney index, and ovary index, with high-frequency electroacupuncture being more effective than low-frequency electroacupuncture. PD patients often experience immune dysfunction and inflammatory responses (38, 56). The spleen, as a crucial immune organ, is commonly assessed by its organ index to reflect the development of immune organs—the larger the spleen mass, the stronger the body’s immune capacity (57). In this experiment, the spleen index of rats in the model group was significantly reduced, while the spleen index in the HF-EA group increased more markedly than in the LF-EA group. This study suggests that high-frequency electroacupuncture can enhance the spleen index, thereby aiding in the restoration and strengthening of impaired splenic immune function.

Although the pathophysiology of dysmenorrhea has not been fully elucidated, current evidence indicates that the pathogenesis of dysmenorrhea is closely related to Prostaglandin F2α (PGF2α) and Prostaglandin E2 (PGE2) (58). PGE2 is a key mediator of inflammatory states and pain; it can dilate blood vessels and relax smooth muscles. In contrast, PGF2α induces constriction of uterine arteries, leading to ischemia and hypoxia, thereby causing menstrual pain (59). Cyclooxygenase (COX) is a critical rate-limiting enzyme in the synthesis of PGE2, existing in two isoforms, COX-1and COX-2, which synergistically participate in PGE2 synthesis. The expression of COX-2 typically requires stimulation by growth factors or inflammatory responses, classifying it as an inducible enzyme. Under pathological conditions (inflammation, injury), inducible COX-2 plays a more significant role in PGE2 synthesis (60). The function of PGE2 depends on the type of its receptor (61). PGE2 receptors, known as E-type prostaglandin receptors (EP receptors), belong to the G protein-coupled receptor family. Each receptor subtype couples with different subunits of heterotrimeric G proteins. There are four subtypes of EP receptors: EP1, EP2, EP3, and EP4 (62). PGE2 mediated by the EP2 receptor also acts to relax blood vessels and inhibit uterine smooth muscle contractions (63). Furthermore, PGE2 mediated by the EP2 receptor can even exacerbate uterine edema and leukocyte recruitment (24). PGE2 is synthesized from arachidonic acid catalyzed by cyclooxygenases COX-1and COX-2 to form PGH2, which is further converted by PGE synthase. The isomerization of the peroxide PGH2 to PGE2 is catalyzed by three different PGE synthases: cytoplasmic PGE synthase (cPGES) and two membrane-bound PGE synthases, mPGES-1 and mPGES-2, Among these isomerases, cPGES and mPGES-2are constitutive enzymes, while mPGES-1 is primarily an inducible isomerase, cPGES utilizes PGH2 produced by COX-1, whereas mPGES-1 utilizes peroxide derived from COX-2, mPGES-2can utilize PGH from both sources and is upregulated in response to various pro-inflammatory stimuli, coinciding with increased expression of COX-2 (64–66).

EA has been shown to alleviate inflammatory pain by more effectively suppressing inflammation. This effect may be mediated through two pathways: firstly, EA activates sympathetic nerve fibers, enhancing the migration of opioid-containing cells to the site of inflammation; secondly, EA triggers the hypothalamic-pituitary-adrenal (HPA) axis, leading to a reduction in COX-2 levels, which in turn interferes with endocannabinoid metabolism and elevates opioid levels at the inflammatory site;furthermore, EA has been reported to decrease COX-2 expression (67, 68), with studies indicating that EA intervention can significantly reduce COX-2 protein expression in rats with primary dysmenorrhea (69). EA has also been shown to modulate prostaglandin levels, increasing PGE2 content and decreasing PGF2α content in the serum and uterine tissue of dysmenorrheic rats (52, 70), as well as reducing the PGF2α/PGE2 ratio (70, 71). Additionally, EA has been found to increase β-EP content (72, 73), effectively inhibiting pain. In the context of post-thoracotomy pain, EA’s analgesic effects may be attributed to its stimulation of endogenous β-EP release and suppression of inflammatory mediators (74). Consistent with these findings, our study demonstrated that both low-frequency and high-frequency EA interventions reduced the levels of COX-2, mPGES-1, PGF2α, and the PGF2α/PGE2 ratio, while increasing the levels of β-EP and PGE2, as well as the protein expression of β-EP and EP2, in dysmenorrheic rats.

The COX-2/mPGES-1/PGE2 pathway plays a crucial role in the development of primary dysmenorrhea by promoting inflammatory responses and oxidative stress (23, 24). COX-2 and mPGES-1 are key regulatory proteins in this pathway. Our study observed an activation of the COX-2/mPGES-1/PGE2 pathway in rats with PD, characterized by increased levels of COX-2, mPGES-1, PGF2α and PGF2α/PGE2 ratio, as well as reduced levels of β-EP and PGE2.Our findings suggest that both low-frequency and high-frequency EA treatments for PD may alleviate uterine spasmodic contractions and improve uterine ischemia and hypoxia by reducing COX-2 and mPGES-1 levels in the serum, uterus, hypothalamus, and spinal cord. This, in turn, suppresses the expression of pro-inflammatory factors and enhances the release of analgesic substances such as β-EP and PGE2, which relax uterine smooth muscle, thereby achieving anti-inflammatory and analgesic effects. These results are consistent with previous literature reports (75, 76).

In summary, both low-frequency electroacupuncture and high-frequency alleviate inflammatory and visceral pain in PD rats by activating the COX-2/mPGES-1/PGE2 signaling pathway. They achieve this by reducing the levels of COX-2, mPGES-1, and PGF2α, as well as the PGF2α/PGE2 ratio, in serum, uterine tissue, hypothalamus, and spinal cord. Additionally, they decrease the protein expression of COX-2 and mPGES-1 in uterine tissue and spinal cord. Conversely, they increase the levels of PGE2 and β-endorphin (β-EP) in serum, uterine tissue, hypothalamus, and spinal cord, along with enhancing the protein expression of EP2 and β-EP. These effects collectively reduce inflammatory and visceral pain in PD rats, while also strengthening their immune and anti-inflammatory functions and alleviating pathological damage to immune organs. The anti-inflammatory and analgesic mechanisms of EA are exerted through both peripheral and central systems, thereby achieving the therapeutic goal of EA for PD. Our findings indicate that high-frequency EA may demonstrate superior anti-inflammatory and analgesic effects compared to low-frequency EA as high-frequency EA more effectively reduces COX-2, mPGES-1, and PGF2α levels, lowers the PGF2α/PGE2 ratio, and suppresses COX-2 and mPGES-1 protein expression, while concurrently elevating both content and protein expression of PGE2 andβ-EP. Our study provides experimental evidence for optimizing the selection of electroacupuncture frequency in the clinical treatment of primary dysmenorrhea.

This study has limitations as well. In the experiment, the spleen index of rats in the model group decreased and was only assessed based on spleen mass; HE staining of the spleen should have been performed. Additionally, the thymus, another immune organ, was not included for HE staining and pathological analysis. These aspects will be further addressed in future research. While our short-term experimental results suggest that high-frequency electroacupuncture may offer certain advantages over low-frequency electroacupuncture in alleviating dysmenorrhea symptoms, it is important to note that the absence of long-term follow-up data and clinical trial validation means that our conclusions in this research are preliminary. Further research, including long-term studies and clinical trials, is necessary to confirm the therapeutic effects of electroacupuncture in treating dysmenorrhea.

## Data Availability

The original contributions presented in the study are included in the article/**Supplementary Material**. Further inquiries can be directed to the corresponding author.
