# Supplementary material for: Effects of low-frequency and high-frequency electroacupuncture pretreatment on the COX-2/mPGES-1/PGE2 pathway in a rat model of cold-coagulation dysmenorrhea
Source: Front Immunol. 2025 Jun 4;16:1563626. doi: 10.3389/fimmu.2025.1563626 (PMC12173867; doi:10.3389/fimmu.2025.1563626)

**Supplemental Figure**


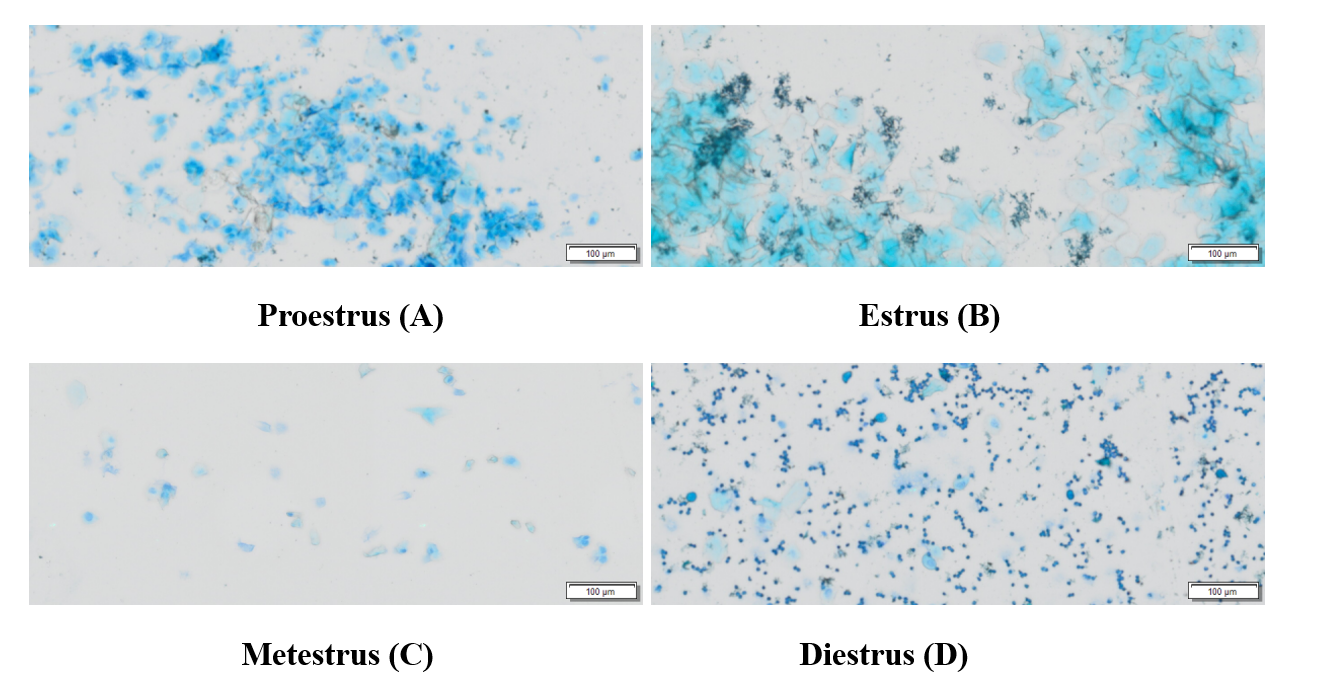


**Supplemental Figure 1. Typical vaginal smears of rats at different stages of the estrus cycle (Scale bar = 100 μm)**


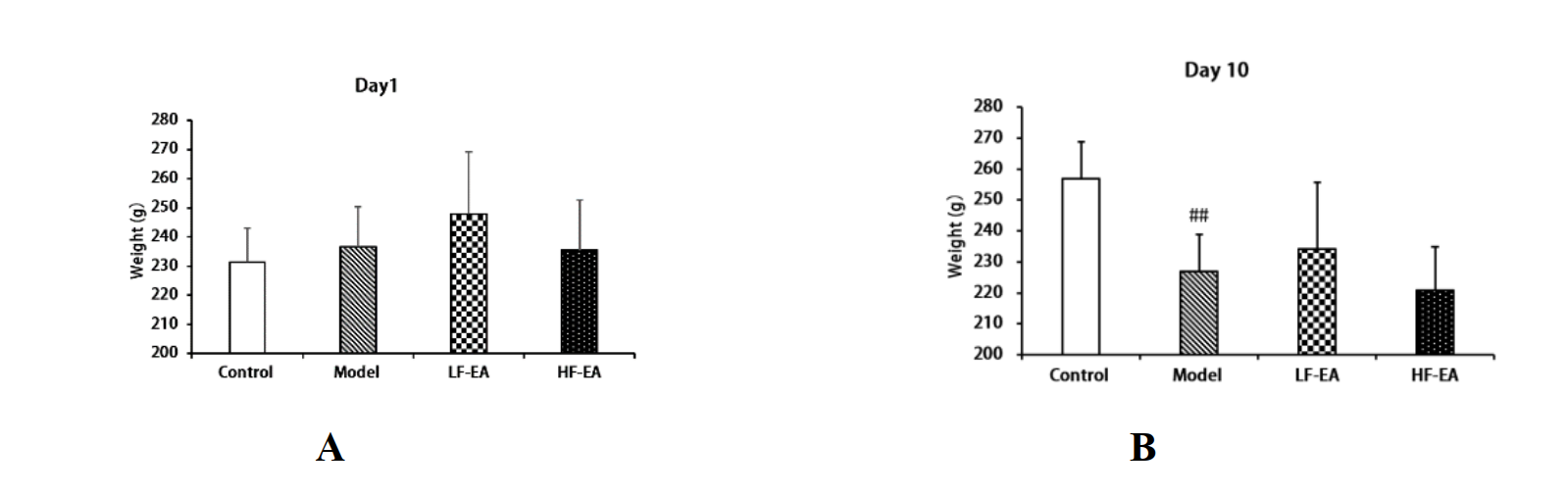
**Supplemental Figure 2. Changes in body weight of rats before and after treatment in each group**


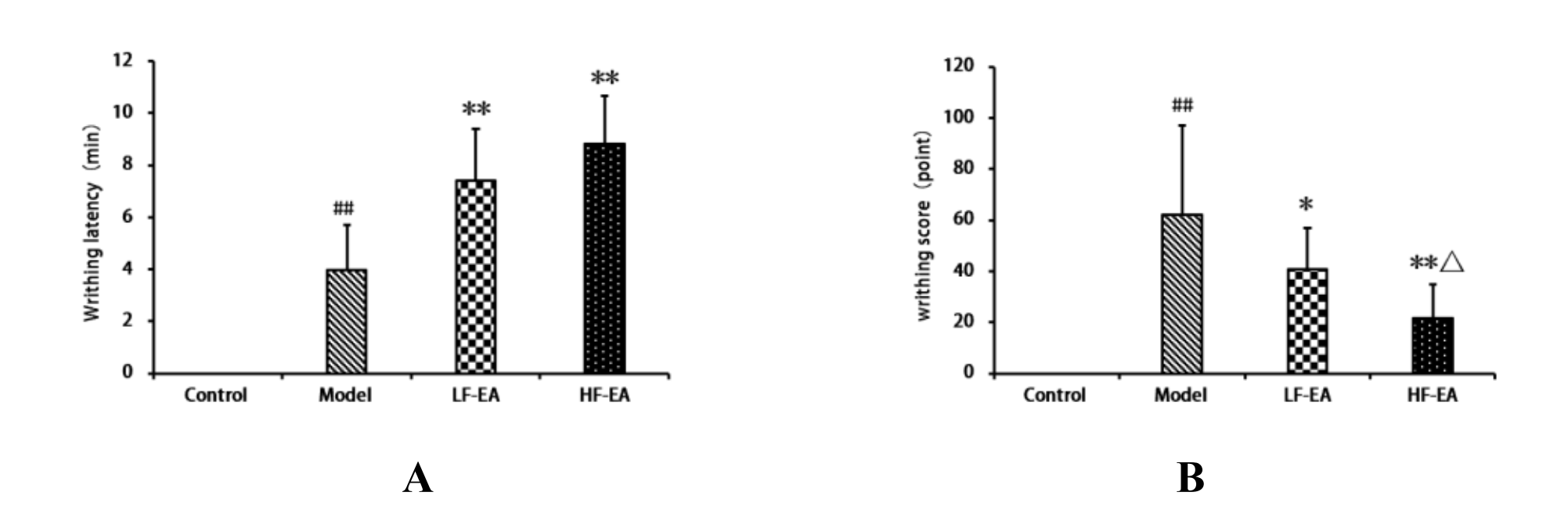
**Supplemental Figure 3. Observation of writhing responses in each group of rats**


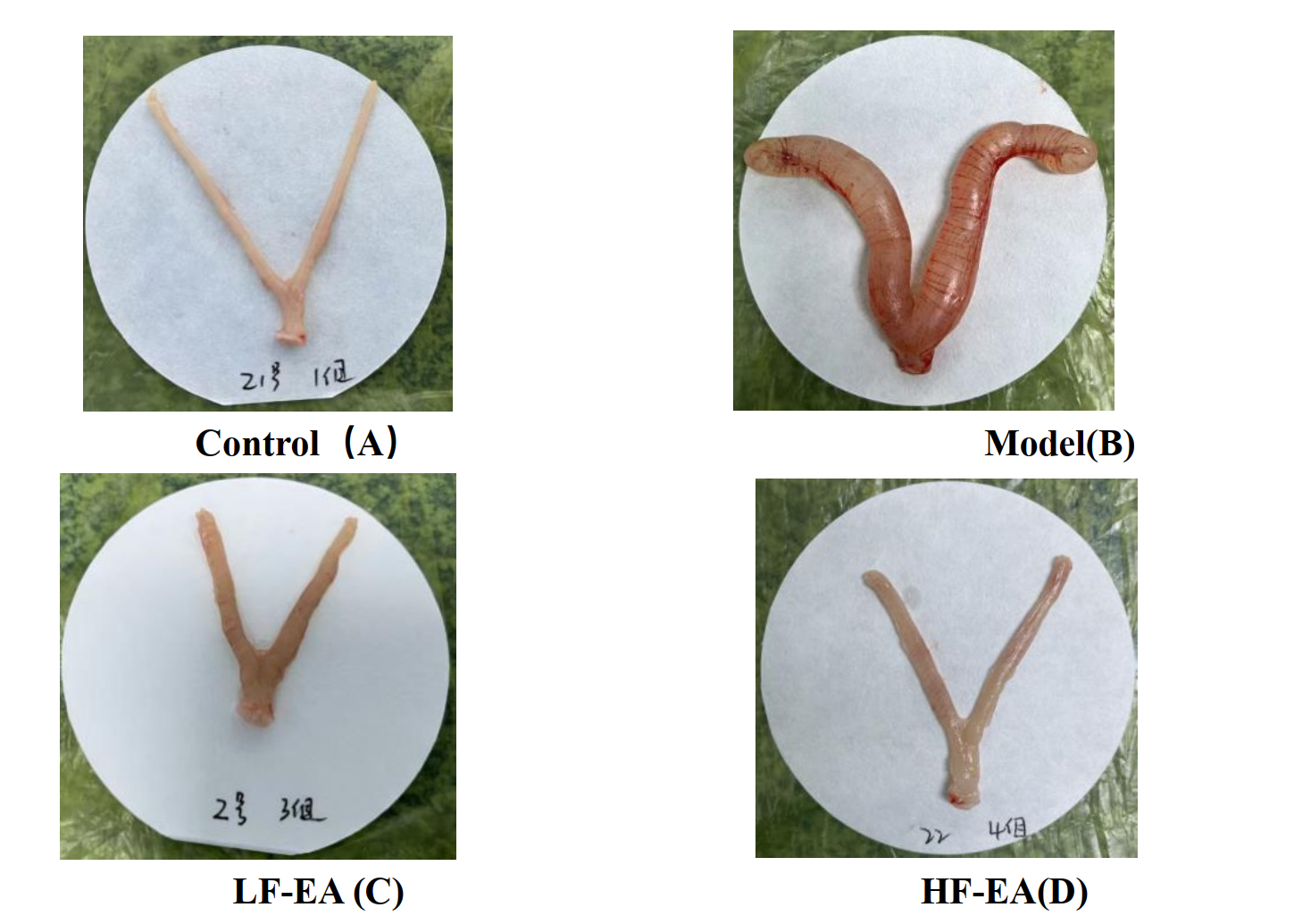


**Supplemental Figure 4. Comparison of uterine morphology in each group of rats**


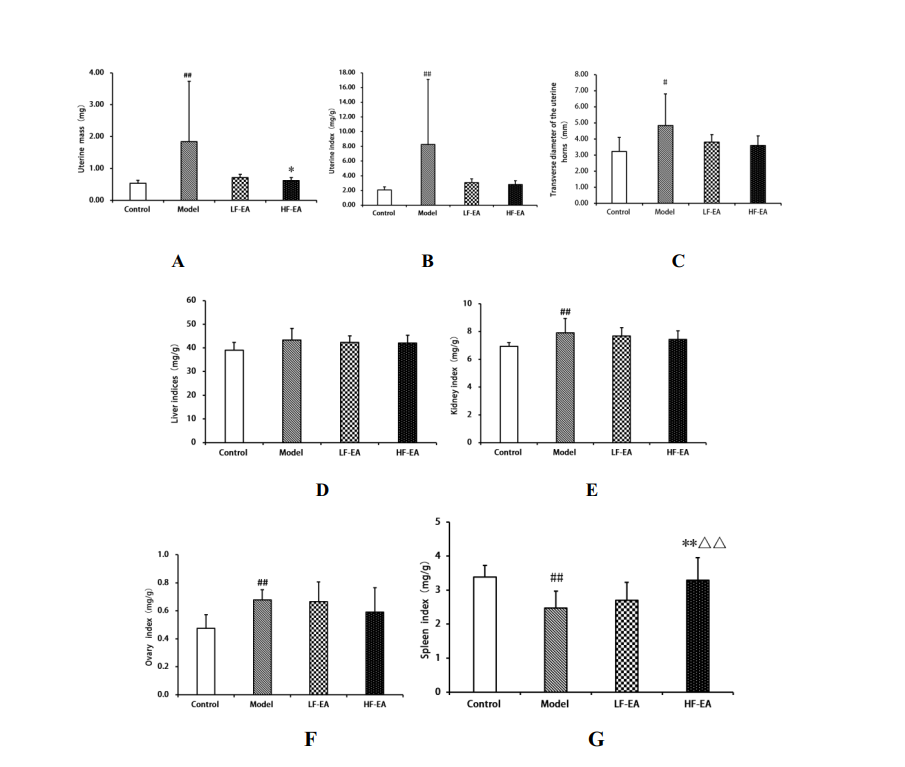


**Supplemental Figure 5. Comparison of uterine mass, uterine index, transverse diameter of the uterine**


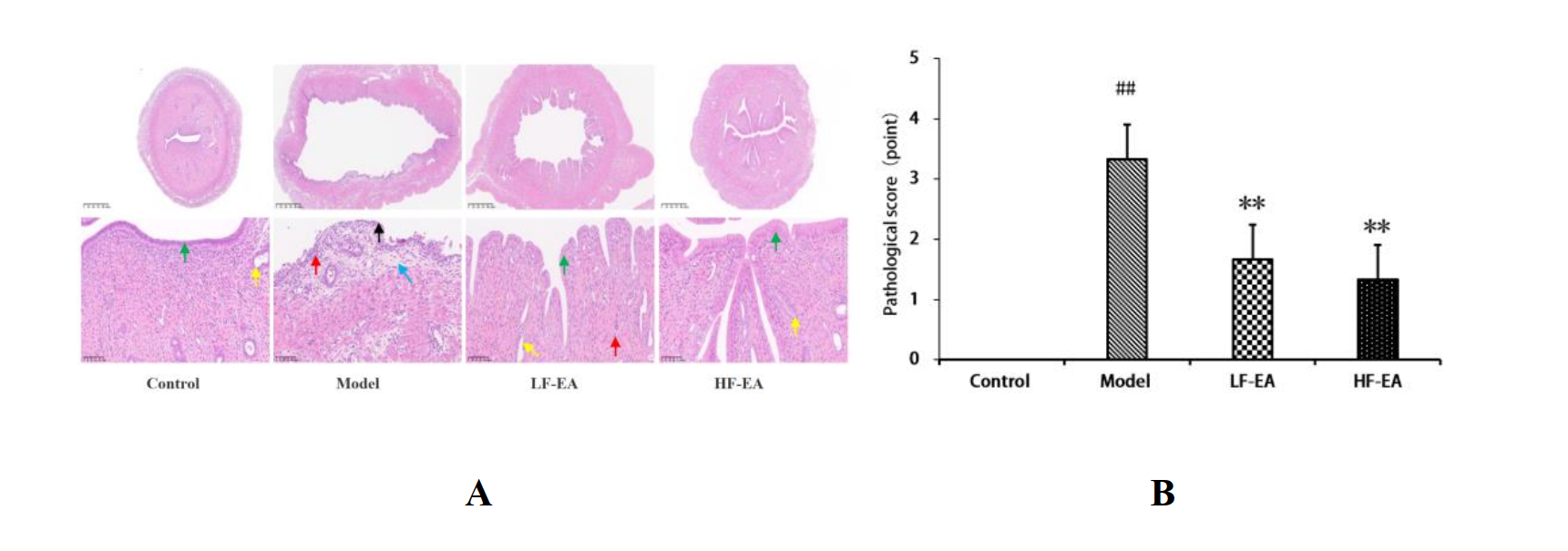


**Supplemental Figure 6. Comparison of uterine tissue histopathology and pathological scores by HE staining among each group of rats**


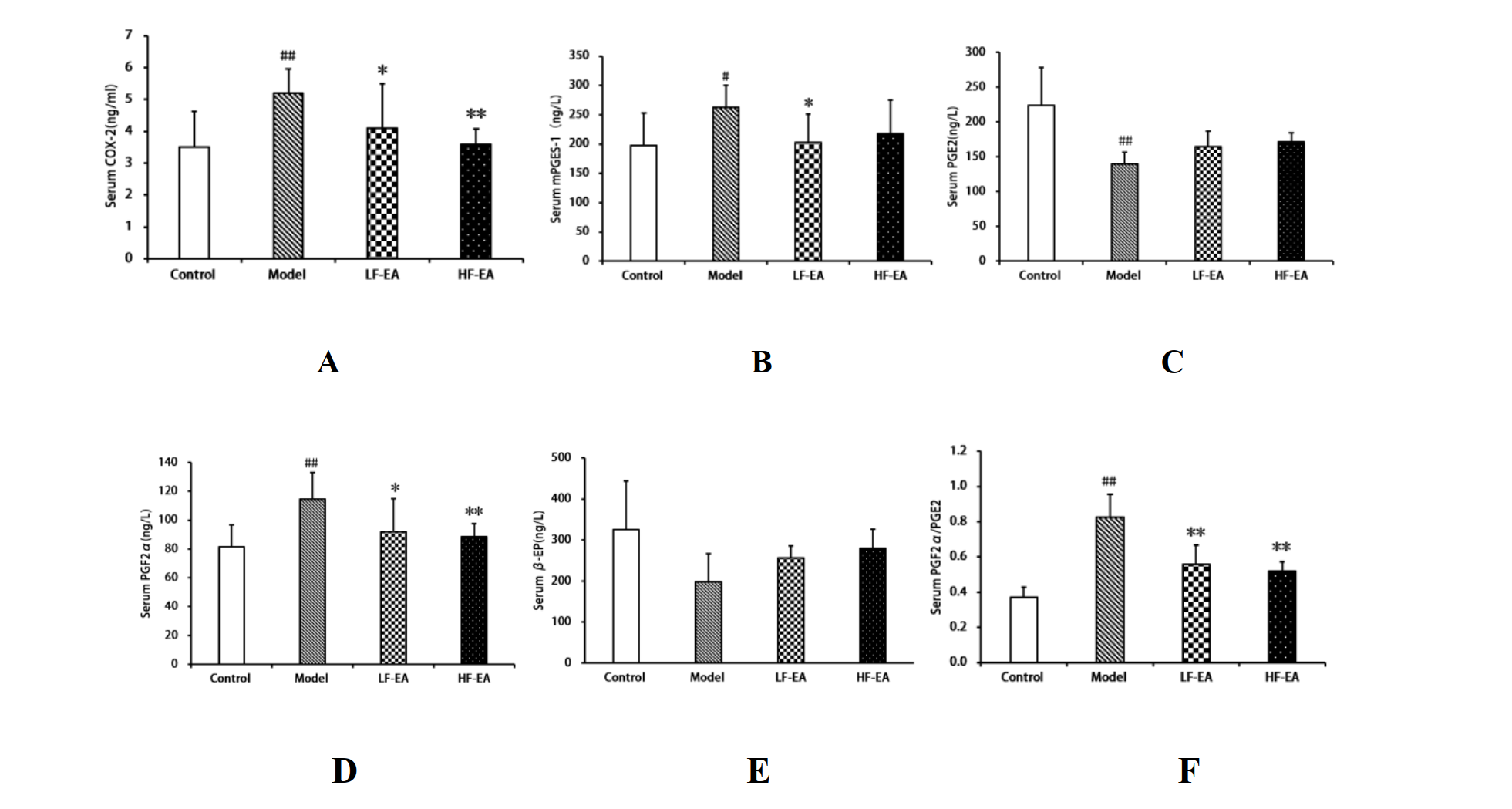
 **Supplemental Figure 7. Comparison of COX-2, mPGES-1, PGE2, PGF2α, β-EP levels, and PGF2α/PGE2 ratio in the serum of rats in each group**


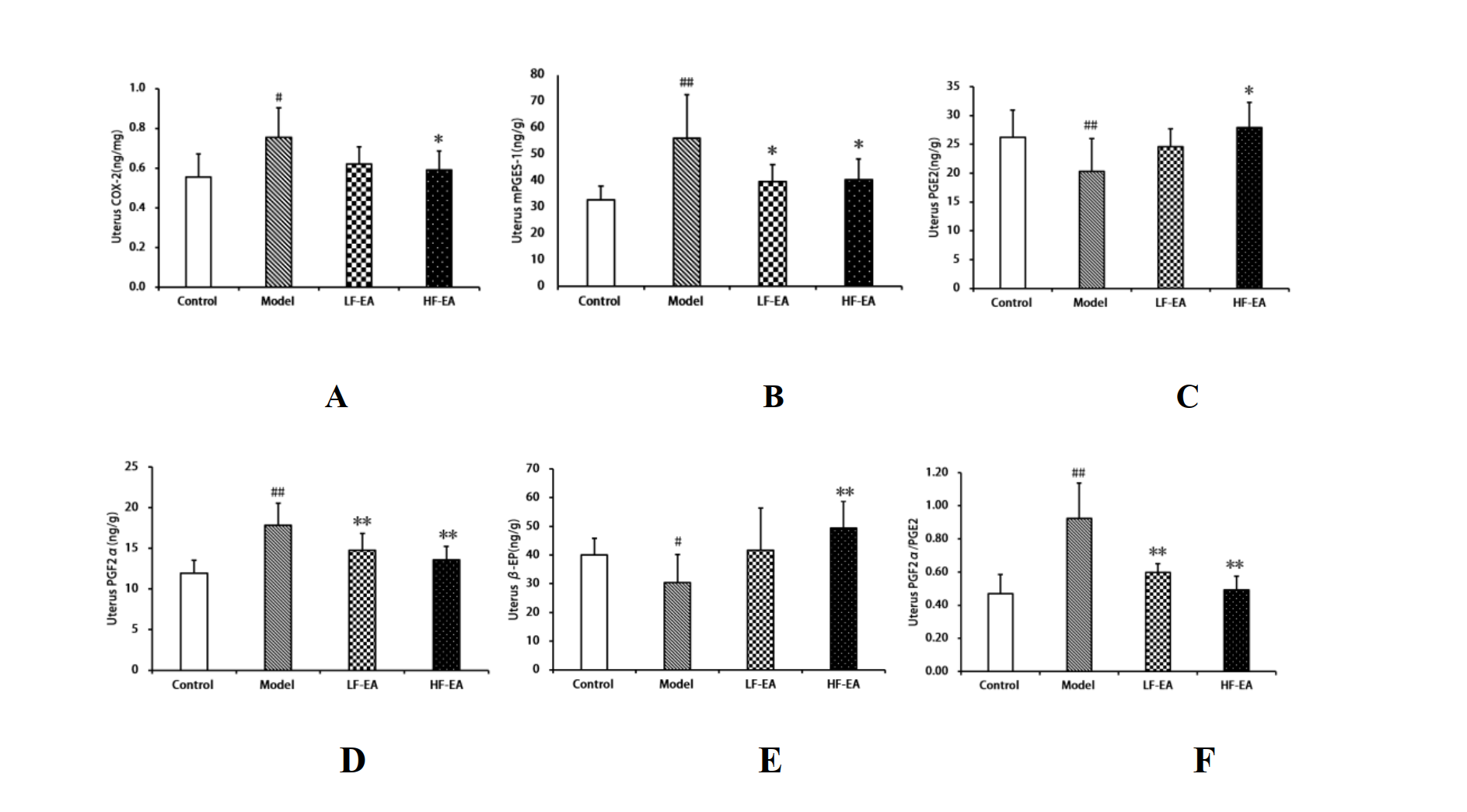


**Supplemental Figure 8. Comparison of COX-2, mPGES-1, PGE2, PGF2α, β-EP levels, and PGF2α/PGE2 ratio in the uterus of rats in each group**


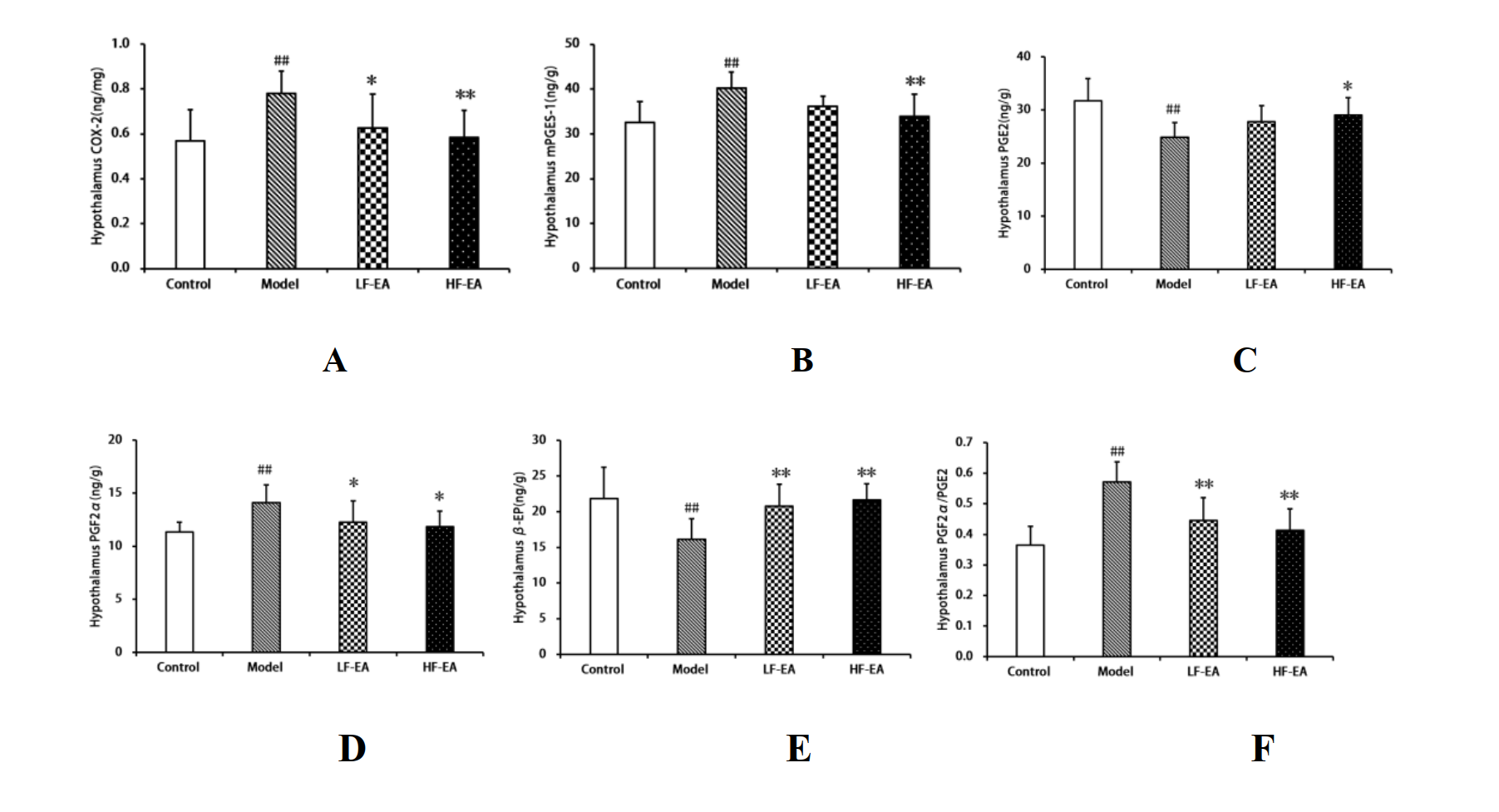
 **Supplemental Figure 9. Comparison of COX-2, mPGES-1, PGE2, PGF2α, β-EP levels, and PGF2α/PGE2 ratio in the hypothalamus of rats in each group**


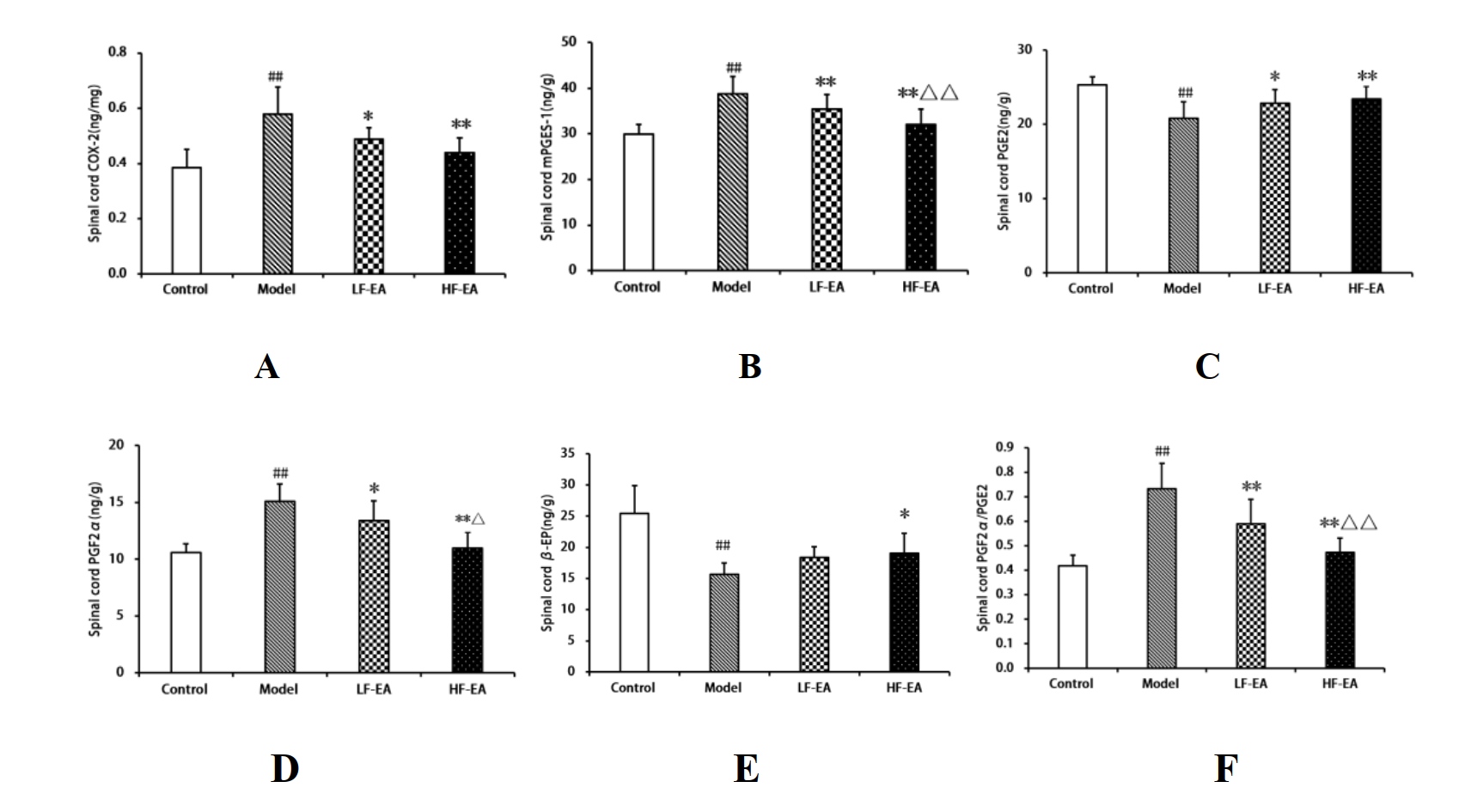
 **Supplemental Figure 10. Comparison of COX-2, mPGES-1, PGE2, PGF2α, β-EP levels, and PGF2α/PGE2 ratio in the spinal cord of rats in each group**

**
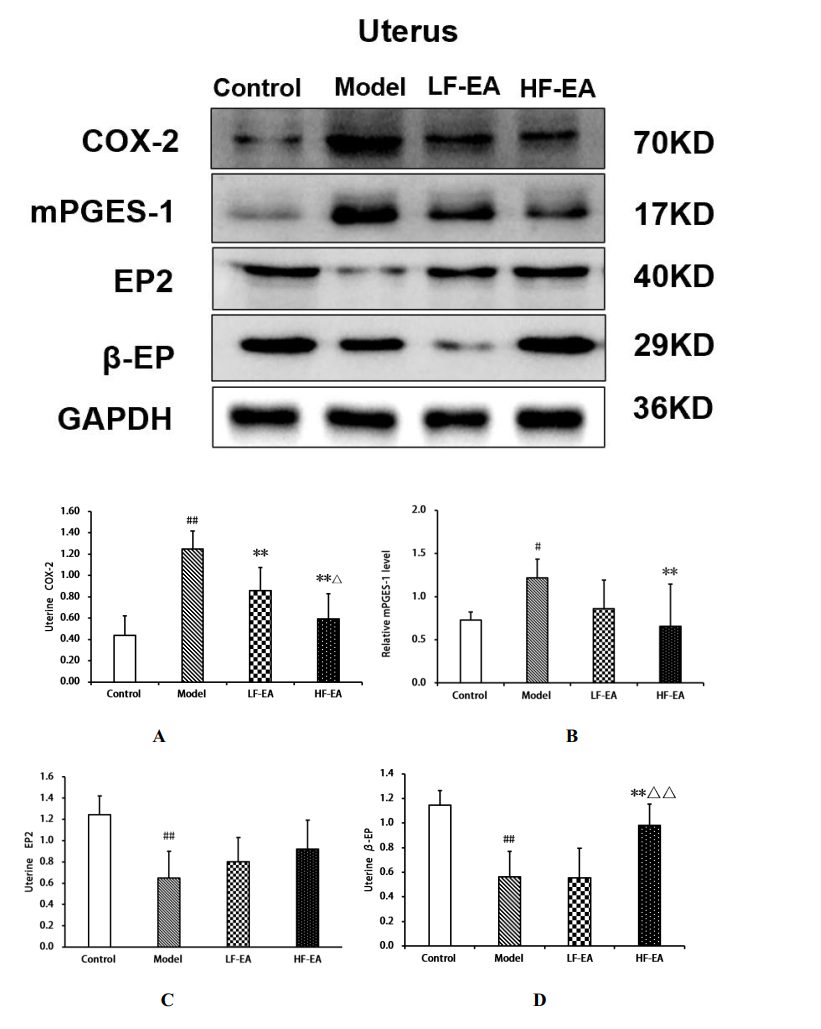
 Supplemental Figure 11. Expression levels of COX-2, mPGES-1, EP2, β-EP proteins in uterine tissue of rats in each group**

**
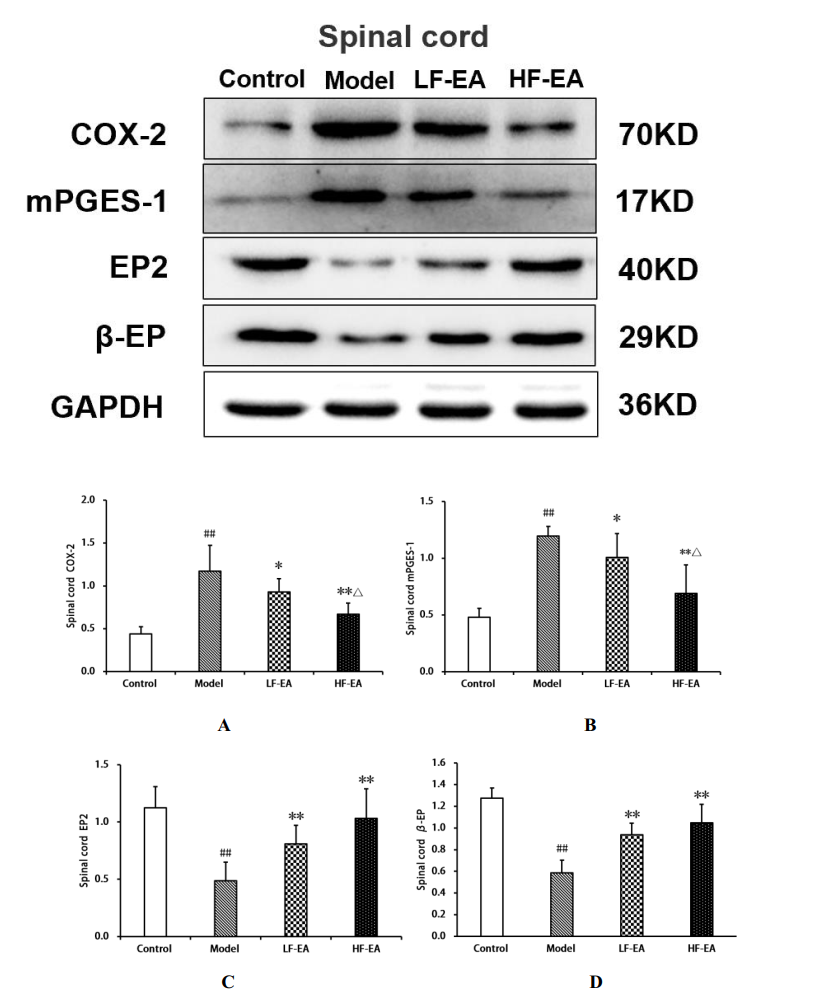
 Supplemental Figure 12. Expression levels of COX-2, mPGES-1, EP2, β-EP proteins in the spinal cord of rats in each group**

**COX-2 in uterine tissue（**repeat the strip diagram of the sample**）**


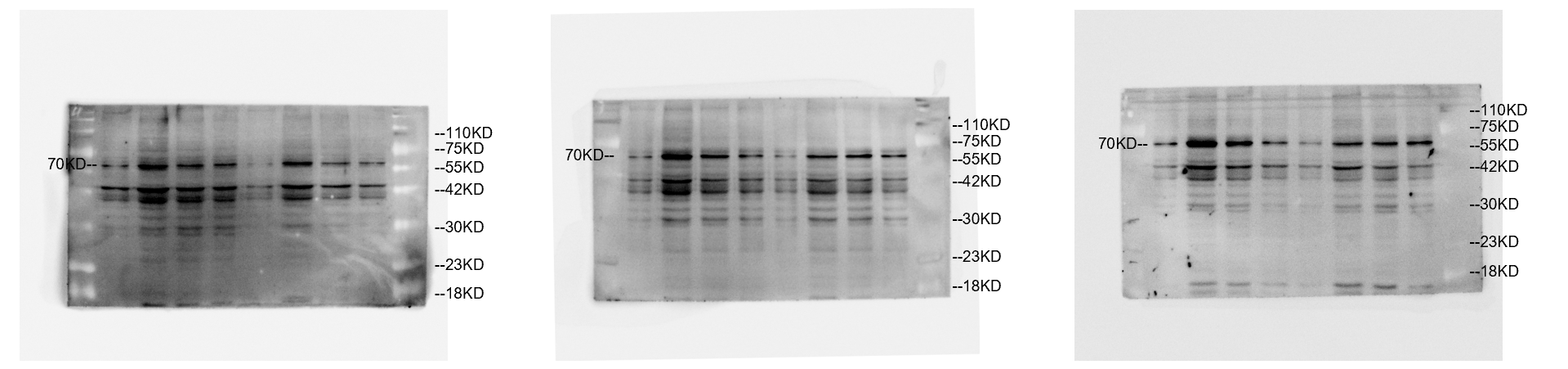


**mPGES-1 in uterine tissue（**repeat the strip diagram of the sample**）**


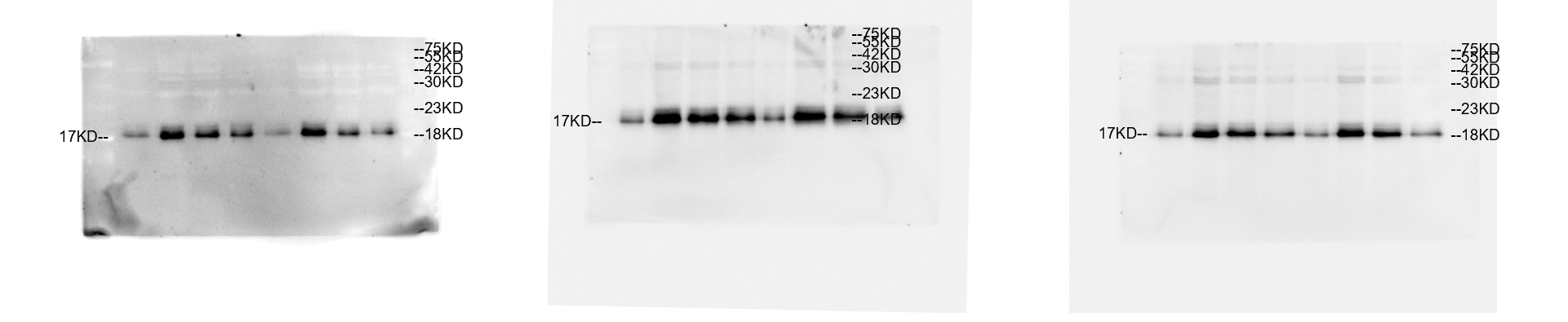


**EP2 in uterine tissue（**repeat the strip diagram of the sample**）**


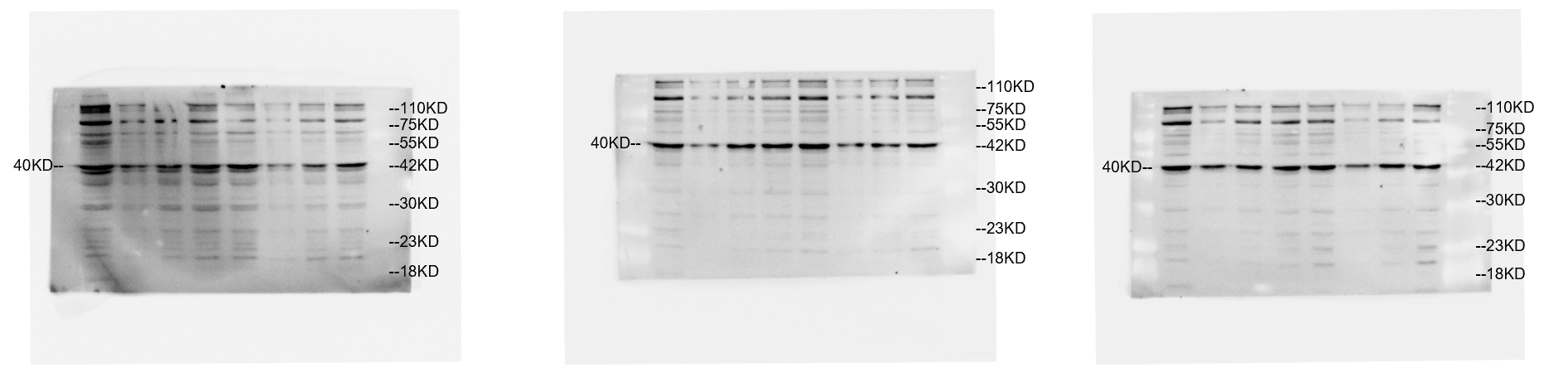


**β-EP in uterine tissue（**repeat the strip diagram of the sample**）**


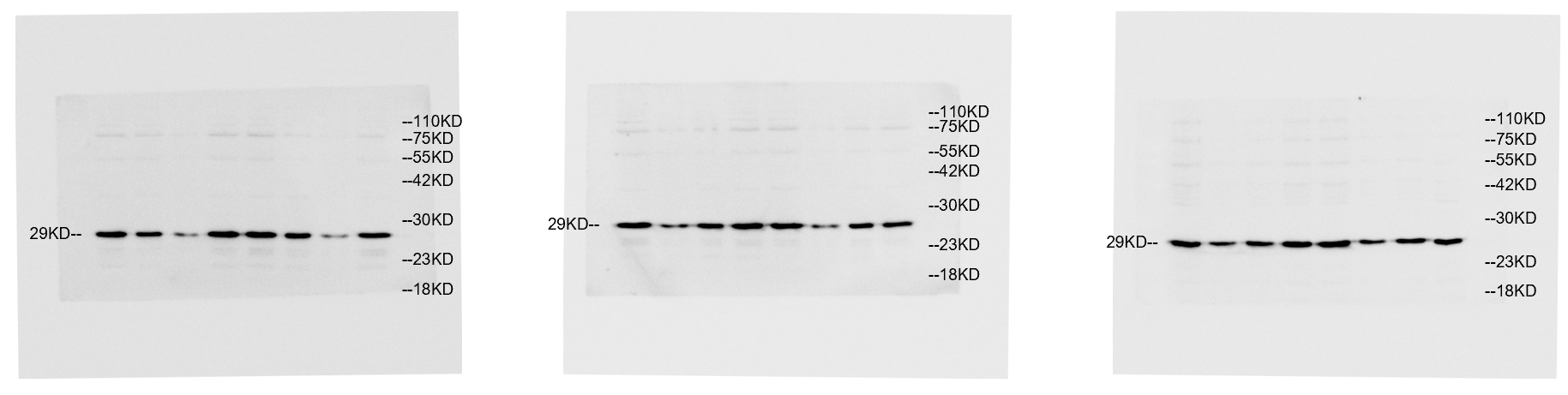


**GAPDH in uterine tissue（**repeat the strip diagram of the sample**）**


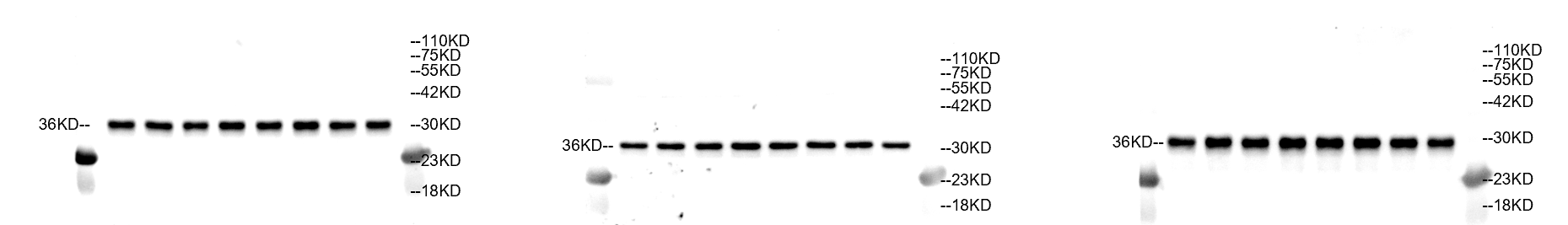


**COX-2 in spinal cord（**repeat the strip diagram of the sample**）**


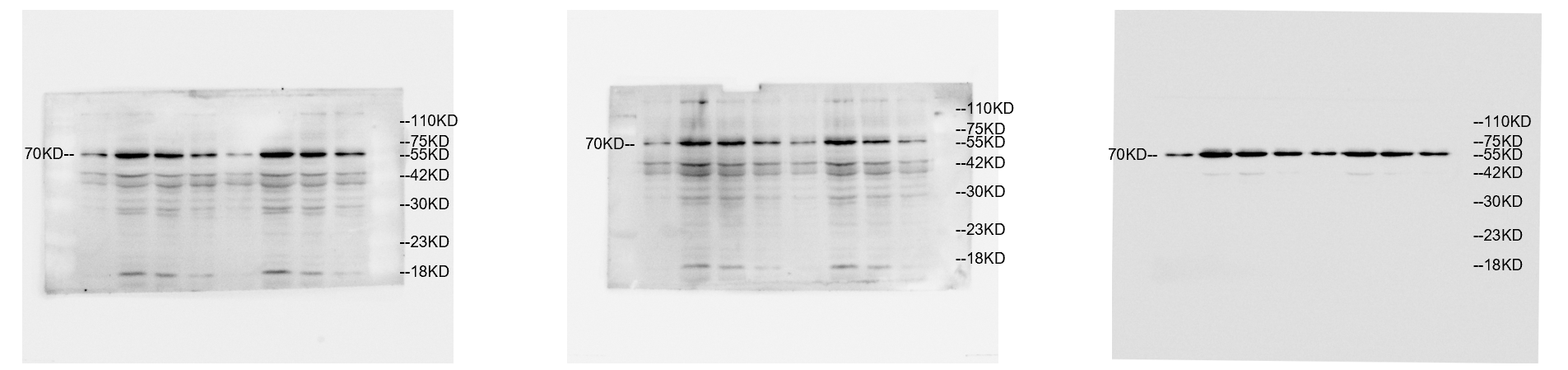


**mPGES-1 in spinal cord（**repeat the strip diagram of the sample**）**


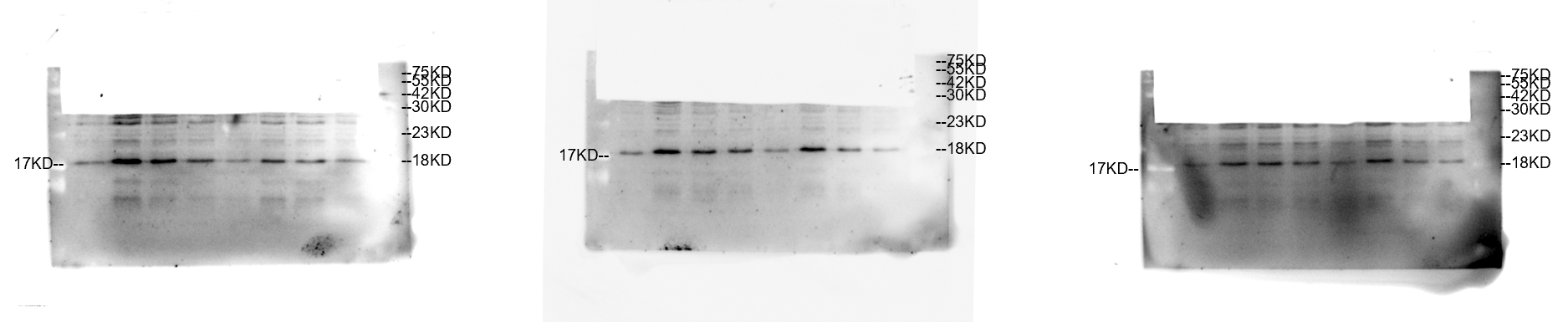


**EP2 in spinal cord（**repeat the strip diagram of the sample**）**


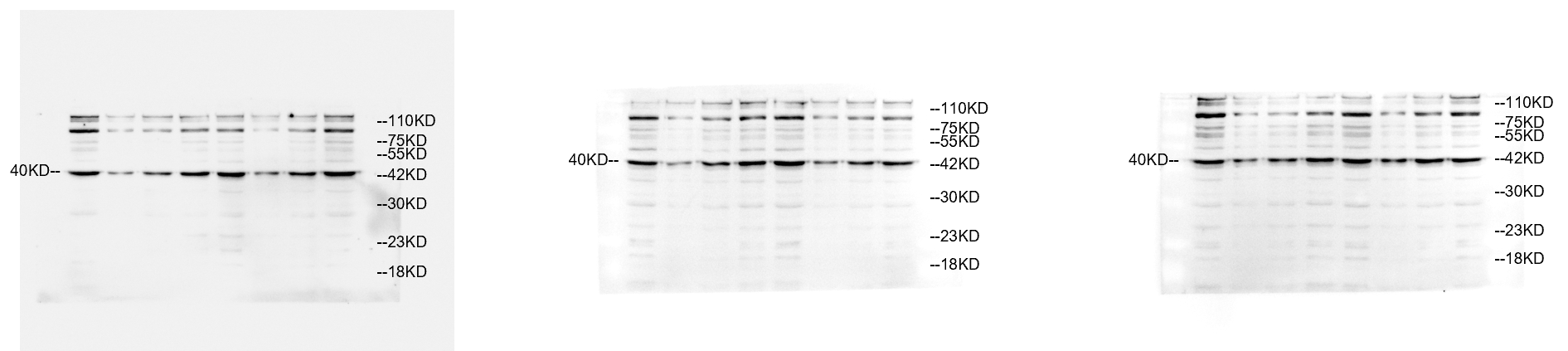


**β-EP in spinal cord（**repeat the strip diagram of the sample**）**


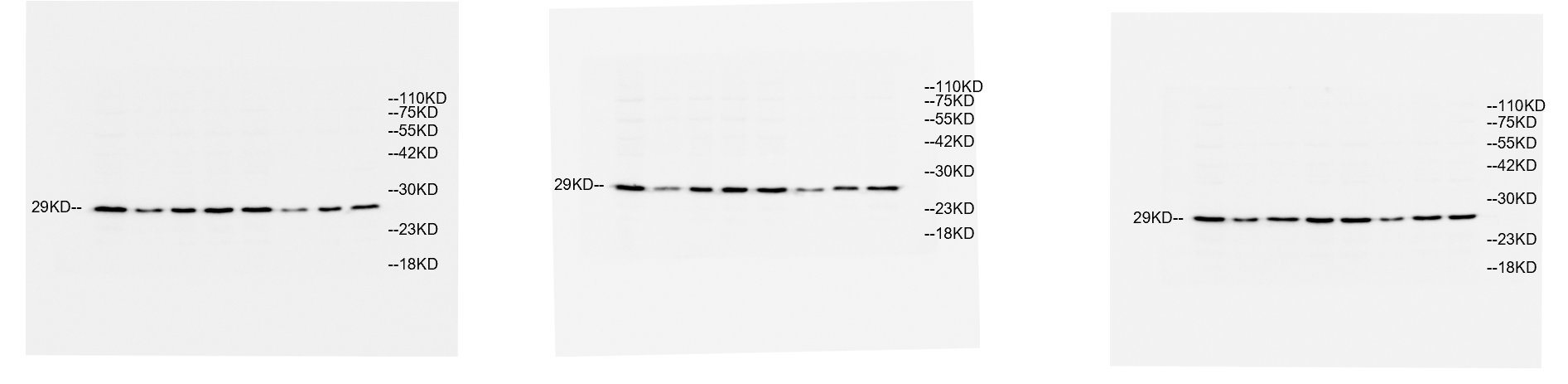


**GAPDH in spinal cord（**repeat the strip diagram of the sample**）**


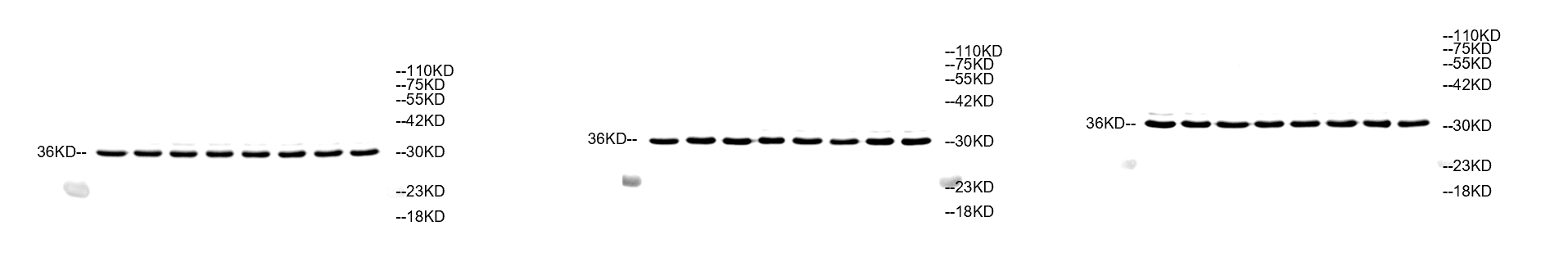

Supplement: Supplementary file 2 [file Table2.docx]
