# Supplementary material for: Effects of low-frequency and high-frequency electroacupuncture pretreatment on the COX-2/mPGES-1/PGE2 pathway in a rat model of cold-coagulation dysmenorrhea
Source: Front Immunol. 2025 Jun 4;16:1563626. doi: 10.3389/fimmu.2025.1563626 (PMC12173867; doi:10.3389/fimmu.2025.1563626)
Supplement: Supplementary file 4 [file Table4.docx]

**Acupuncture Procedures**

**Acupuncture Point Localization:**

Guanyuan (CV 4): Located approximately 25 mm below the umbilicus.

Sanyinjiao (SP 6): Positioned 10 mm directly above the medial malleolus of the hindlimb.

Ciliao (BL 32): Situated in the second posterior sacral foramen, roughly between the posterior superior iliac spine and the midline of the back.

**Needling Techniques:**

CV 4: Insert the needle perpendicularly to a depth of 5 mm. Employ a uniform lifting-thrusting and twirling manipulation technique, characterized by slow, even, and gentle force. Ensure that the force, amplitude, and frequency of lifting and thrusting, as well as left and right rotation, are equal. Maintain a twirling angle between 90° and 180°, and minimize the lifting-thrusting amplitude to facilitate "de qi" (the sensation of needle stimulation). The needle is not retained after manipulation.

BL 32: Insert the needle at a 45° angle to a depth of 10-15 mm. Apply the same uniform lifting-thrusting and twirling manipulation technique for 30 seconds.

SP 6: Insert the needle perpendicularly to a depth of 5 mm and perform the uniform lifting-thrusting and twirling manipulation technique.

The experimental researchers must be proficient in needling techniques to minimize discomfort for the rats and adjust the needle depth as needed based on the rat's reactions to maintain comfort and safety.

When removing the needle, do so slowly and steadily to prevent bleeding or infection.

After completing the needling session, observe the rats for any signs of adverse reactions or complications. Provide appropriate care and monitoring as needed.
